# Supplementary material for: Using a Resuscitation-Based Simulation Activity to Create an Interprofessional Education Activity for Medical, Nursing, and Pharmacy Students
Source: MedEdPORTAL. 2020 Dec 11;16:11054. doi: 10.15766/mep_2374-8265.11054 (PMC7732132; doi:10.15766/mep_2374-8265.11054)
Supplement: Supplementary file 1 — Simulation Case Template.docxAgenda.docDebriefing Guide.docFaculty Training PowerPoint.pptxHospital Tech.docxMedication List.docxPrebrief Information.docxMedication Administration Record.docxFaculty Assessment Tool.xlsxStudent Questionnaire.docx [file mep_2374-8265.11054-s001.zip › _Educational_Summary_Report_11054.pdf]

MedEdPORTAL® The AAMC Journal of Teaching and Learning Resources

Original Publication Open Access

Using a Resuscitation-Based Simulation Activity to Create an Interprofessional Education Activity for Medical, Nursing, and Pharmacy Students

M. Tyson Pillow, MD, MEd, Catherine L. Hatfield, PharmD, Rebecca Aulbach, PhD, Rita Dello Stritto, PhD, Peggy Landrum, PhD, Suzanne Scheller, MS, Joel Purkiss, PhD, Anne C. Gill, DrPH*

*Corresponding author: gill@bcm.edu

Abstract

Introduction: To achieve high-quality, patient-centered care, teaching programs across health professions must prepare their learners to work in effective teams. We created a simulation activity to formatively assess interprofessional objectives in graduating medical, nursing, and pharmacy students. This simulation also gave learners an opportunity to practice clinical airway resuscitation skills. **Methods:** The simulation featured a decompensating adult asthmatic with a chief complaint of shortness of breath and a final diagnosis of severe asthma exacerbation and respiratory failure. Students completed a prebrief to formulate a plan and then interacted with a mannequin. Faculty led a debriefing and completed assessments of the team's performance. The students completed a questionnaire assessing their own and the team's performance. **Results:** Four sessions were held over a 2-year period. A total of 91 graduating students participated in the activity: 33 from Baylor College of Medicine, 26 from University of Houston College of Pharmacy, and 28 from Texas Woman's University Nelda C. Stark College of Nursing. Postsession questionnaire data demonstrated very good overall team performance and good individual performance. Student comments demonstrated an understanding of the importance of teamwork and thoughtful reflection on their own areas for improvement. All students rated the activity as valuable and effective. Multirater assessments of the students found that most met three of the four objectives. **Discussion:** This activity allows for real-time formative assessment with a focus on roles, communication, and managing difficult situations. The debriefing demonstrates the students' understanding of interprofessional goals in providing effective patient-centered care.

Keywords
Interprofessional Education, Respiratory Distress, Resuscitation, Simulation, Nursing Students, Pharmacy Students, Emergency Medicine

Educational Objectives

By the end of the session, participants will be able to:

1. Demonstrate respect for the unique cultures, values, roles/responsibilities, and expertise of other health professions.
2. Communicate with team members to clarify each member's responsibility in executing components of a treatment plan or public health intervention.
3. Use respectful language appropriate for a given difficult situation, crucial conversation, or interprofessional conflict.
4. Reflect on individual and team performance for opportunities to improve.

Citation:
Pillow MT, Hatfield CL, Aulbach R, et al. Using a resuscitation-based simulation activity to create an interprofessional education activity for medical, nursing, and pharmacy students. *MedEdPORTAL*. 2020;16:11054. https://doi.org/10.15766/mep_2374-8265.11054

Introduction

The *Core Competencies for Interprofessional Collaborative Practice* promote interprofessional learning and teamwork with the goal of improving patient-centered care.[1,2] Interprofessional education (IPE) is an accreditation requirement for many disciplines[3-5] but can be problematic for nonyoked or stand-alone institutions. Baylor College of Medicine has partnered with other local health professions schools to find innovative methods for IPE activities. Many of these activities utilize different training formats, including didactics, problem-based learning sessions, standardized patients (SPs), and simulation. This specific activity was developed to offer a developmentally appropriate, culminating or capstone clinical IPE activity to more fully prepare students to enter the workforce and/or the

Copyright © 2020 Pillow et al. This is an open-access publication distributed under the terms of the Creative Commons Attribution license.

next phase of their training. The objectives were adapted from the *Core Competencies for Interprofessional Collaborative Practice.*[1,2]

A review of the literature revealed several articles supporting the benefits of interprofessional training using simulation and other teaching modalities.[6-8] While these articles are convincing, comprehensive details to implement such training are lacking. A review of *MedEdPORTAL* publications revealed multiple IPE offerings,[9-11] including but not limited to stroke management, pediatric emergencies, patient safety training, intraoperative arrhythmias, and maternal cardiac arrest. Wilson and Vorvick[12] offer a somewhat similar IPE simulation for dyspneic patients in a hospitalized setting; however, our resuscitation-based simulation activity is unique and differs in many ways. First, our scenario design is different, with a focus on simulation of a critically ill patient where student teams perform independently. They receive no prep before, or prompting during, the case. The students must quickly evaluate an undifferentiated patient while incorporating IPE principles. The case has been designed and refined to be within the limits of medical knowledge but still challenge students to apply that knowledge in real time in a team-based setting. Student performance dictates the path of the case rather than the case following a specific progression and resolution. Therefore, if the students manage the patient correctly and in a timely fashion, the mannequin is more stable before the final deterioration of the case. Pauses and delays in management and decision-making are considered part of the assessment, and the students receive no prompting or interventions to keep the case moving. In contrast, the training designed by Wilson and Vorvick includes preparation by the students on the specific chief complaint, training in IPE with activities, and direct interventions or huddles as needed to guide the performance during the session. The patient in that simulation is also less critical and stabilizes during the scenario. Another unique aspect of our simulation is that students are challenged to perform their own medication administration. A crash cart with medications is provided, as well as all necessary equipment, to administer critical medications in real time. In almost all instances, real oxygen and oxygen delivery equipment have been used, and students have been required to push medications instead of simply verbalizing the action. Sanseau and colleagues[13] published a collection of pediatric cases, but the simulation case described here is in an adult and much more critical than their published cases. No other publications specifically for IPE simulation of adult patients with acute respiratory distress including unique roles for the disciplines of nursing, pharmacy, and medicine were identified. Furthermore, many of the publications we did find describe a guided experience for learners who assume their roles in a fairly prescribed context where major care decisions have already been made. Our experience is completely immersive in that the students make management decisions and see the effects of their decisions in real time.

For 2 years prior to implementing this activity, a “disaster day” activity was conducted that focused on the care of the walking wounded in a disaster scenario. These sessions were well received, but students did not fully comprehend or grasp the interprofessional objectives desired for the activity. The novelty and urgency of the scenario overwhelmed many of the learners, and the interprofessional aspect of the simulation went largely unnoticed. Therefore, the activity was redesigned both to be more specific to their clinical skills and to emphasize teamwork. To underscore the teamwork, a debriefing guide was created to foster reflection; this guide introduced an IPE assessment modeled on a validated rubric[14] to assess individual and team performance during the simulation. The activity was changed from disaster day to a crisis-management activity to capture the stressors associated with sick patients and the necessity of teamwork. The first version of the crisis-management activity involved SPs as both the patient and the family member, but students were easily distracted by the interactions with the SPs. Students work with SPs in another simulation published in *MedEdPORTAL* related to patient and interprofessional communication.[15] Therefore, to maintain a clear focus on teamwork within the constraints of a 20-minute simulation, the family member was removed, the SP was replaced with a mannequin, and communication occurred through a headset in the final version of the experience.

This IPE activity is unique because it immerses the student team in the patient’s care. Rather than assume the role of other care providers and react to actions external to them, students in this formative assessment interact directly with the patient (mannequin) for history, physical exam, and ongoing assessment, as well as with each other. The prebrief, activity, and debrief center around the actual team’s and the individuals’ actions in caring for a patient. The case makes the interprofessional collaboration internal rather than external, and the richness of the experience is captured and reinforced in the debriefing activity immediately after caring for the patient.

Methods

Development

The goal was to immerse students in a moderately high-fidelity, real-time, realistic resuscitation that did not require responses too

Copyright © 2020 Pillow et al. This is an open-access publication distributed under the terms of the Creative Commons Attribution license.

far in excess of their current clinical knowledge. The case was carefully designed by faculty from each discipline and revised to be at an appropriate level for all of the students according to their discipline. We conducted four sessions of this scenario for teams consisting of senior medical students (fourth-years), senior nursing students, and fourth-year pharmacy students. Medical students were assigned to the simulation as one of several breakout options during their fourth-year capstone course. Nursing and pharmacy faculty assigned students enrolled in senior-level courses. All students had completed the majority of their required clinical experiences prior to the activity and had exposure to communication and team training in prior IPE activities.[11,15] Medical students had exposure observing the care of sick patients in their emergency medicine training. Nursing and pharmacy students had previously participated in an IPE communications skills course, and all pharmacy students had previous TeamSTEPPS training.[16] Approximately 65 faculty from three institutions (Baylor College of Medicine, University of Houston College of Pharmacy, and Texas Woman's University Nelda C. Stark College of Nursing) participated in one or more of the sessions. Faculty roles include clinical expert (physician and nurse practitioners), debriefer, assessor, observer and case manager. Faculty could elect to serve in multiple roles as appropriate and were trained accordingly. Emergency medicine faculty who participated in the activity were trained to run the mannequin prior to the activity. All faculty who participated in the activity received debriefing training and assessment instructions from the dean of IPE at Baylor College of Medicine. The training consisted of the simulation case overview (Appendix A), the agenda (Appendix B), and the debrief (Appendix C), which was conducted both in person and via PowerPoint webinar (Appendix D) as necessary. To ensure psychological safety of the debriefing process while addressing critical mistakes, faculty were instructed to use yellow cards (yellow notepaper) to capture clinical errors that needed to be corrected. Any concerns identified on the yellow cards were addressed in private with the team or individual by faculty from their discipline before the team or individual student was dismissed from the simulation. The major actions of the case included continuing nebulized albuterol and ipratropium, administering steroids, identifying a magnesium sulfate allergy, creating a differential diagnosis, and identifying when to initiate bag valve mask respirations.

Equipment/Environment

Equipment necessary to run the activity included the following:

- Full-sized hospital bed.
- Full-sized adult simulation mannequin.
- Simulated monitor system with adjustable vital signs.
- Intravenous fluid bag and drip set.
- Oxygen tank (or other source of oxygen, simulated or real).
- Nasal canula.
- Non-rebreather mask.
- Nebulizer mask.
- Bag valve mask.
- Fully stocked simulated crash cart (critical simulated medications noted):
  - Albuterol nebulizer capsules.
  - Ipratropium nebulizer capsules.
  - Prednisolone vials.
  - Magnesium sulfate.
  - Epinephrine 1:1000 vials.
- Syringes (1-cc, 3-cc, 5-cc, and 10-cc with appropriate needles or needle-less systems).
- Optional: intubation equipment (laryngoscope blade, endotracheal tube, 10-cc syringe).

The initial setup included an IV with fluids connected and the mannequin on nasal cannula, sitting up in the bed in a tripod position.

Personnel

Each session was referred to as a wave of students. The personnel necessary to run each wave included the following:

- IPE activity managers: one to two faculty or staff who ensured the flow of the entire activity, including preparation/setup of equipment; flow of students, faculty, and staff; and any other details of the session.
- Case manager: trained facilitator for each team responsible for the progression of the case, who answered student questions during the activity and managed the patient's vital signs.
- Hospital technician: trained facilitator who participated in the activity directly with the student team to facilitate the case. This person managed technical problems in real time, assisted the team with medication administration if they were not able to do it themselves, and pointed out changes in the patient's condition when prompting of the team was needed.
- Debriefer: faculty trained to lead the team debrief after the activity.
- Observer: faculty trained to observe the entire activity and complete the faculty assessment forms.

Implementation

The simulation activity took place in the Texas Woman's University Nelda C. Stark College of Nursing simulation center.

Copyright © 2020 Pillow et al. This is an open-access publication distributed under the terms of the Creative Commons Attribution license.

Prebriefing occurred in a nearby conference room, and debriefing happened in the same area as the activity. Students worked in teams of three, one from each discipline. Each team participated in one resuscitation activity, and two to four teams participated in the activity at the same time.

Setup included positioning the mannequin sitting up in a tripod position in the hospital bed. An IV line was connected to fluid and taped to the mannequin. The mannequin had an allergy band that indicated an allergy to iodine but omitted an allergy to magnesium sulfate. Patient monitors were tested and set to normal for the brief tour of the simulation space for each wave.

Each room was led by a case manager who had been trained by a simulation faculty member. The role of the case manager was to interact with the team and answer questions through the mannequin via a headset. The case manager also controlled the progression of the case, adjusted the monitors to reflect treatment decisions, and provided imaging and lab results, as applicable. Each room also had a hospital tech (Appendix E) trained by a simulation faculty whose role was to passively support the team with any technical skills or real-time problems encountered in the case. For example, the hospital tech assisted the team in placing the patient on oxygen and drawing up medications in order to prevent these tasks from hindering the flow of the case. The hospital tech first started by giving the team a tour of the simulation area for their case and instructions on interacting with the patient. There was also a list of drugs available in the crash cart during the case (Appendix F).

The case involved the patient, Mr. Smith, who had been admitted from the emergency room for an asthma exacerbation. While being managed by the admitting team, the patient was started on treatments and signed out to the student team. Prior to going to the bedside, the student team received the sign-out via a written summary of the patient's course, vital signs, and other pertinent information and formulated an initial plan (Appendix G). Next, the team moved to the simulation room and cared for the patient. A medication allergy to magnesium sulfate was embedded in the case and identified on the medication administration record (Appendix H) but not on the patient's allergy band. The case manager gave the medication administration record directly to the pharmacy student at the beginning of the case (within the first 2 minutes). Care of the patient proceeded until the patient was stabilized or 20 minutes had passed. The simulation then ended, and the students transitioned to a group debriefing led by trained faculty.

Assessment

Data were collected on both team and individual performance from a faculty observation tool (Appendix I) and from a student questionnaire (Appendix J) at the completion of the activity. The faculty assessment tool was based on a modified McMaster-Ottawa Scale for assessing performance in an interprofessional team observed structured clinical encounter.[14] In parallel, students completed an online questionnaire that assessed team performance, individual performance, and satisfaction with the activity.

Debriefing

Each student team had one to three faculty observers (different disciplines) who observed the prebriefing and the simulation, either led or took notes during the debriefing, and completed a student assessment form. Faculty were identified and recruited from each institution with an interest and/or background in simulation or IPE. All faculty assessors and observers received debriefing training and assessment instructions from the dean of IPE at Baylor College of Medicine. The training consisted of a review of the case and discussion of each page of the debriefing guide and was conducted both in person and via webinar with PowerPoint slides. Subsequent facilitators were paired with senior faculty and first observed and then performed a debriefing, receiving immediate feedback after the activity.

Results

The first session was conducted in January 2018, with subsequent larger sessions in March 2018, October 2018, and February 2019. In total, 91 students participated in the activity: 33 medical students, 28 nursing students, 26 pharmacy students, and four who did not disclose their profession. The students were randomly assigned to teams consisting of one student from each discipline. Seven of the teams across the activities had four students due to extra students being available. Of the 91 participants, 87 completed the questionnaire, for a response rate of 96%. Among respondents, 38% were medical students, 30% were pharmacy students, and 32% were nursing students.

Postactivity student data on team performance revealed that overall, students felt they had achieved the goals of the activity (Table 1). One hundred percent of participants agreed or strongly agreed that they respected the expertise of other health professionals. Ninety-seven percent agreed or strongly agreed that they worked well with the members of the health care team. Eighty-six percent agreed or strongly agreed that they utilized the abilities of team members to optimize care. Eighty percent agreed or strongly agreed that they used effective

Copyright © 2020 Pillow et al. This is an open-access publication distributed under the terms of the Creative Commons Attribution license.

Table 1. Student Self-Assessment of Performance on Objectives[a]

Topic	Agree or Strongly Agree: No. (%)	Disagree or Strongly Disagree: No. (%)
Respected the expertise of other health professionals (objective 1).	90 (100)	0 (0)
Worked well with other members of the team (objective 1).	90 (100)	0 (0)
Utilized abilities of team members to optimize patient care (objective 2).	77 (86)	13 (14)
Used effective communication tools and techniques (objective 2).	72 (80)	18 (20)
Interacted in a respectful manner when dealing with conflict (objective 3).	69 (88)	9 (12)
Recognized how individual knowledge, skills, and experience and the hierarchy of the team contributed to effective collaborative care (objective 4).	80 (95)	5 (5)

[a]Item-level number varies due to nonresponse to some assessment items.

communication. Ninety-four percent agreed or strongly agreed that they interacted in a respectful manner when dealing with conflict. Overall, 73% agreed or strongly agreed that their team performance was good. The results of the student questionnaire reveal that the students felt they performed well as a team (Figure 1) and accomplished the team-based goals of the activity. The following quotations are representative of student comments about team performance:

- “I thought this was a really great scenario in understanding each other’s roles.”
- “Team worked well together. Each member valued the other’s knowledge and experience.”
- “Communicated well and overcame obstacles despite not taking the most effective route.”

Interestingly, the students were a bit more critical of themselves as individuals (Figure 2) even when participating in a team that they determined to be successful. Only 55% rated their individual performance as good or very good overall, in contrast to 73% who rated their team’s performance as good or very good overall. The following quotations are representative of student comments about individual performance:

- “I need to be more assertive in my role.”
- “I could have communicated way better with my team.”
- “I could have made better decisions that related to my role, but I feel like the case went well and was surprised at how I was confident in my abilities in the moment.”

When asked to comment on areas of the activity that made it effective for learning, many students indicated that the scenario was realistic and tense and that they appreciated the “tons of feedback.” When asked for areas of improvement, most of their comments related to clearer instructions and more time in the planning. Overall, the students valued the activity highly, with many of them asking for more opportunities like this in the curriculum.

Faculty assessments of the student performance data are reported in Table 2. Data were collected from the faculty after each session. Subsequently, statistical analysis was performed to examine for interrater reliability. Since students were assessed

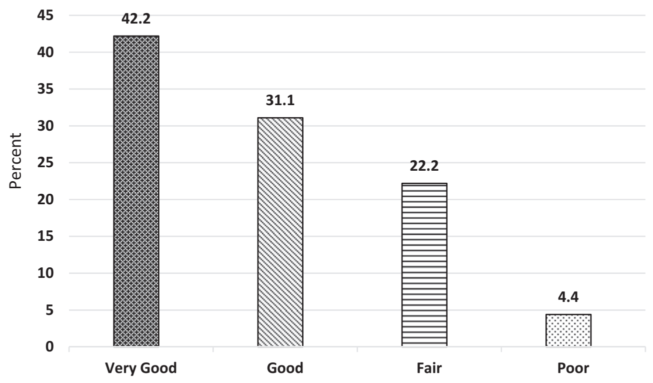

Figure 1. Students’ self-assessment of team performance ($N = 90$).

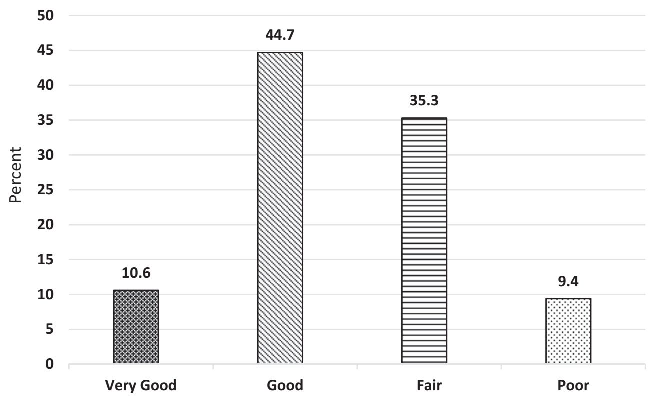

Figure 2. Students’ self-assessment of their own individual performance ($N = 85$).

Table 2. Faculty Ratings of Student Performance ($N = 311$)[a]

Topic	No.	*M*[b]	Mode[b]
Communicates with team members to clarify responsibilities.	292	1.29	1
Uses respectful language appropriate for a given situation.	308	1.55	2
Reflects on individual and team performance.	306	1.70	2
Demonstrates respect for the unique cultures of other professions.	303	1.82	2

[a]Each student was assessed by multiple faculty, so the number of ratings is greater than the number of participating students.
[b]Assessment scale: 0 = *not done*, 1 = *needs improvement*, 2 = *done*.

Copyright © 2020 Pillow et al. This is an open-access publication distributed under the terms of the Creative Commons Attribution license.

by varying numbers of faculty from a pool of available raters, we used randomization to create a data set appropriate for interrater reliability analysis. We randomly selected two faculty scores given to each student using a random number generation feature in Excel (Microsoft Office 2013), so each student had results from only one pair of raters. This process yielded 79 students with paired scores. Cronbach's alpha can be used as a measure of interrater reliability in contexts where many raters are providing scores, as was the case here.[17] For the paired assessments, each provided by two various assessors from an overall pool of 31 raters, $\alpha = .549$. This would generally be interpreted as an indication of insufficient interrater reliability; an alpha level of .7 has typically been considered an indication of minimal reliability.[18] We note, however, that additional analysis provides some evidence for a modest degree of interrater reliability. There was a moderate but statistically significant correlation between the two ratings ($R = .381$, $p = .001$), and a paired-sample t test found that students' scores from their randomly selected assessors did not differ significantly ($p = .203$). Overall, we interpret these results as indicating we could do more to increase the interrater reliability of this assessment tool before using it in a summative manner, but with some minimal evidence of rater agreement, we are comfortable with its continued use in our current formative assessment context.

As summarized in Table 2, the majority of students did meet three of the four objectives. The objective that appeared to need the most improvement was "Communicate with team members to clarify each member's responsibility in executing components of a treatment plan or public health intervention." Faculty rated the students on this objective during both the prebrief and the activity. Not only did most students from all disciplines need improvement but this was also the objective where an assessment of not done at all was seen the most ($n = 27$).

Discussion

This immersive crisis-management simulated learning activity allowed senior-level students to practice advanced clinical skills and reinforced the importance of teamwork. The assessment was an internal activity that put the students in a situation where they had to care for the patient and make clinical decisions within their domains. A common limitation of IPE activities is that students are able to remain external to the events, meaning that they take the role of the person who makes a mistake or must break bad news but who has not taken care of the patient originally. In contrast, our type of simulation places the student team into a first-person role where they must make decisions and then hold themselves and their team members accountable for those decisions. Additionally, our case was specifically designed to challenge the students without overwhelming them in terms of their current clinical knowledge or skills. It was important to the leadership team to give the students a solvable problem. When the students became stuck on clinical questions or technical skills, the case manager or hospital tech immediately helped them to navigate the situations so that the focus would be on the interprofessional skills. Similarly, a crisis was used at a very specifically designed level. This patient was sick enough to require immediate attention but did not decompensate into complicated pathways. Nor did the patient die in the scenario.

One of the themes that arose from the activity was the idea of roles, particularly in regard to tasks and leadership. Even though the pharmacy students had participated in TeamSTEPPS early in their training, there was no discernible difference in their performance compared to the other learners. In terms of tasks, there was no clear understanding of other disciplines' scope with regard to multiple tasks during the case. This phenomenon was seen with student and faculty expectations of other disciplines. For example, the case required a nebulizer to be placed on the patient. It was very common to see the nursing student assume the pharmacist would do it, the pharmacy student assume the nurse would do it, and the medical student unsure of how to perform or troubleshoot the task at all. In contrast, one frequent underlying theme was that the medical student was assumed to be team leader. This played out in a variety of ways, including situations where the student stepped up to lead but was not challenged when making mistakes. On the other end of the spectrum were medical students who froze and nursing or pharmacy colleagues who stepped up and led. These interactions clearly displayed the delicate balance between shared decision-making and definitive leadership in critical situations.

The activity received a significant amount of informal feedback, improving the quality and strength of the scenario and ensuring the students would be presented with a realistic situation similar to what they would theoretically begin to face in just a few short months. Even when the teams had difficulties, many students wanted another try and stated that they knew they could improve with more practice. Multiple students commented that they would value more and earlier experiences with this type of training in their respective curricula. Finally, students commented on the value of actually being able to work in a team to understand other disciplines. Up to that point, the large majority of them had seen other disciplines interact with each other but mostly had seen disciplines remaining in their own silos. This activity gave

Copyright © 2020 Pillow et al. This is an open-access publication distributed under the terms of the Creative Commons Attribution license.

them an opportunity to work as a team outside of those silos and understand their colleagues much better.

Multiple challenges were discovered when implementing this activity. As it was essentially a moderately high-fidelity scenario, specific space and equipment were needed to deliver it to students. The activity has been successfully delivered to almost 100 students across the disciplines, but delivering it consistently to all students across the three institutions every year will take more space and equipment. Obviously, there are also financial costs associated with this type of simulation activity. Some of those costs were mitigated by using a mannequin rather than SPs. Another challenge was coordinating schedules across the three institutions. In particular, the nursing school graduated a class of nurses every 6 months, necessitating at least two sessions per year to capture all of the nursing students. Lining up the offerings to find the least common multiple will be a challenge for the future.

Perhaps the most interesting challenge was discovering students with significant skill or knowledge gaps late in their training. These gaps could range from misunderstandings of mechanisms and indications of common drugs to the most common problem of putting on oxygen and/or nebulizers incorrectly. While this IPE activity provided a formative assessment, its relatively late timing in the curricula of all three institutions created a dilemma. Therefore, the use of yellow cards was implemented. Yellow cards were simply blank sheets of yellow paper where faculty could write down errors in clinical or team performance. Errors in team performance were discussed during the debrief. Individual student errors were discreetly addressed after the session by taking the student aside to correct a knowledge deficit or practice the skill.

One lesson learned during the implementation of this IPE activity is that sometimes students were unable to move forward due to not fully understanding the assumptions or expectations of the simulated environment. Initial efforts to ameliorate this situation were to improve the orientation to the simulation pod by explicitly asking the students to touch and feel the bed, mannequin, and equipment. However, it was not possible to anticipate every clinical contingency, and the addition of the tech as a trusted confederate who could manage the technical or logistical aspects of the case, (e.g., “X-ray has been called, lab results are ready, anesthesia is tied up in a case”) helped the students focus on the IPE aspects rather than the systems and procedure issues.

Another lesson learned was that some faculty left items on the assessment form blank. Reasons for this varied from momentary distractions to not being sure how to answer. Because the form allowed for only three options (done, needs improvement, or not done), we will add an additional column labeled “not able to assess.”

The first limitation of the activity is that there is only one case. The difficulty level and stress created by the case are of critical importance, and each new case will need the same effort and attention to detail to make sure it also works to optimize the experience. Second, in the case’s current iteration as a single formative assessment, there has been a desire across students and faculty to want to improve team performance further, but no follow-up opportunities are currently being offered to do so. Finally, students have been reluctant to call upon the hospital tech to help them, even though that has been explained and reinforced as the tech’s role.

In conclusion, we offer a unique resuscitation-based simulation activity for IPE among medical students, nursing students, and pharmacy students. This learning experience and formative assessment opportunity create a realistic collaborative experience that students enjoy and that is reinforced by faculty observations and debriefing to emphasize the targeted objectives. Moving forward, all three institutions have verbalized a goal of making the activity a required part of their curricula. Thus, more cases fitting the specific needs and goals that drove creation of this activity will have to be created, and ways to incorporate this type of immersive training earlier in the curricula will be discussed.

Appendices

A. Simulation Case Template.docx
B. Agenda.doc
C. Debriefing Guide.doc
D. Faculty Training PowerPoint.pptx
E. Hospital Tech.docx
F. Medication List.docx
G. Prebrief Information.docx
H. Medication Administration Record.docx
I. Faculty Assessment Tool.xlsx
J. Student Questionnaire.docx

All appendices are peer reviewed as integral parts of the Original Publication.

Copyright © 2020 Pillow et al. This is an open-access publication distributed under the terms of the Creative Commons Attribution license.

M. Tyson Pillow, MD, MEd: Associate Professor, Department of Emergency Medicine, Baylor College of Medicine; Medical Director, Simulation and Standardized Patient Program, Baylor College of Medicine

Catherine L. Hatfield, PharmD: Clinical Professor, University of Houston College of Pharmacy

Rebecca Aulbach, PhD: Assistant Professor, Texas Woman's University Nelda C. Stark College of Nursing

Rita Dello Stritto, PhD: Professor, Texas Woman's University Nelda C. Stark College of Nursing

Peggy Landrum, PhD: Clinical Professor, Texas Woman's University Nelda C. Stark College of Nursing

Suzanne Scheller, MS: Associate Clinical Professor, Texas Woman's University Nelda C. Stark College of Nursing

Joel Purkiss, PhD: Assistant Professor, Department of Medicine, Baylor College of Medicine; Assistant Dean of Evaluation, Assessment, and Education Research, Baylor College of Medicine

Anne C. Gill, DrPH: Professor, Department of Pediatrics, Baylor College of Medicine; Assistant Dean of Interprofessional Education, Baylor College of Medicine

Disclosures
None to report.

Funding/Support
None to report.

Prior Presentations
Pillow MT, Livingston L, Teal CR, Gill A, Nelson EA. Disaster day vs. crisis management: two institutions' experiences to answer the call for interprofessional education. Poster presented at: Southern Group on Educational Affairs Regional Meeting; April 25, 2015; Charlotte, NC.

Ethical Approval
Reported as not applicable.

References

1. Interprofessional Education Collaborative. *Core Competencies for Interprofessional Collaborative Practice: Report of an Expert Panel*. Interprofessional Education Collaborative; 2011.
2. Interprofessional Education Collaborative. *Core Competencies for Interprofessional Collaborative Practice: 2016 Update*. Interprofessional Education Collaborative; 2016.
3. Liaison Committee on Medical Education. *Functions and Structure of a Medical School: Standards for Accreditation of Medical Education Programs Leading to the MD Degree*. Liaison Committee on Medical Education; 2020.
4. Accreditation Council for Pharmacy Education. *Accreditation Standards and Key Elements for the Professional Program in Pharmacy Leading to the Doctor of Pharmacy Degree*. Accreditation Council for Pharmacy Education; 2015.
5. Commission on Collegiate Nursing Education. *Standards for Accreditation of Baccalaureate and Graduate Nursing Programs*. Commission on Collegiate Nursing Education; 2018.
6. Kelm DJ, Ridgeway JL, Gas BL, et al. Mindfulness meditation and interprofessional cardiopulmonary resuscitation: a mixed-methods pilot study. *Teach Learn Med*. 2018;30(4):433-443. https://doi.org/10.1080/10401334.2018.1462186
7. Onan A, Simsek N, Elcin M, Turan S, Erbil B, Deniz KZ. A review of simulation-enhanced, team-based cardiopulmonary resuscitation training for undergraduate students. *Nurse Educ Pract*. 2017;27:134-143. https://doi.org/10.1016/j.nepr.2017.08.023
8. Gilfoyle E, Koot DA, Annear JC, et al.; Teams4Kids Investigators; Canadian Critical Care Trials Group. Improved clinical performance and teamwork of pediatric interprofessional resuscitation teams with a simulation-based educational intervention. *Pediatr Crit Care Med*. 2017;18(2):e62-e69. https://doi.org/10.1097/PCC.0000000000001025
9. McDonough KA, White AA, Odegard PS, Shannon SE. Interprofessional error disclosure training for medical, nursing, pharmacy, dental, and physician assistant students. *MedEdPORTAL*. 2017;13:10606. https://doi.org/10.15766/mep_2374-8265.10606
10. Forstater A, Levinson M, Bellot J, Hess M, Spandorfer J. Patient safety symposium: issues, analyses, prevention. *MedEdPORTAL*. 2013;9:9637. https://doi.org/10.15766/mep_2374-8265.9637
11. Gill AC, Cowart JB, Hatfield CL, et al. Patient safety interprofessional training for medical, nursing, and pharmacy students. *MedEdPORTAL*. 2017;13:10595. https://doi.org/10.15766/mep_2374-8265.10595
12. Wilson S, Vorvick L. Dyspnea in a hospitalized patient: using simulation to introduce interprofessional collaborative practice concepts. *MedEdPORTAL*. 2016;12:10488. https://doi.org/10.15766/mep_2374-8265.10488
13. Sanseau E, Reid J, Stone K, Burns R, Uspal N. Pediatric simulation cases for primary care providers: asthma, anaphylaxis, seizure in the office. *MedEdPORTAL*. 2018;14;10762. https://doi.org/10.15766/mep_2374-8265.10762
14. Lie D, May W, Richter-Lagha R, Forest C, Banzali Y, Lohenry K. Adapting the McMaster-Ottawa Scale and developing behavioral anchors for assessing performance in an interprofessional team observed structured clinical encounter. *Med Educ Online*. 2015,20(1):26691. http://dx.doi.org/10.3402/meo.v20.26691
15. Kusnoor AV, Gill AC, Hatfield CL, et al. An interprofessional standardized patient case for improving collaboration, shared accountability, and respect in team-based family discussions. *MedEdPORTAL*. 2019;15:10791. https://doi.org/10.15766/mep_2374-8265.10791

Copyright © 2020 Pillow et al. This is an open-access publication distributed under the terms of the Creative Commons Attribution license.

16. TeamSTEPPS 2.0. Agency for Healthcare Research and Quality. December 2012. Updated June 2019. Accessed April 7, 2020. https://www.ahrq.gov/teamstepps/instructor/index.html
17. Stemler SE. A comparison of consensus, consistency, and measurement approaches to estimating interrater reliability. *Pract Assess Res Eval*. 2004;9:4. https://doi.org/10.7275/96jp-xz07
18. Nunnally JC. *Psychometric Theory*. 2nd ed. McGraw-Hill; 1978.

Received: April 20, 2020
Accepted: August 24, 2020
Published: December 11, 2020

Copyright © 2020 Pillow et al. This is an open-access publication distributed under the terms of the Creative Commons Attribution license.
