# Supplementary material for: Using a Resuscitation-Based Simulation Activity to Create an Interprofessional Education Activity for Medical, Nursing, and Pharmacy Students
Source: MedEdPORTAL. 2020 Dec 11;16:11054. doi: 10.15766/mep_2374-8265.11054 (PMC7732132; doi:10.15766/mep_2374-8265.11054)
Supplement: Supplementary file 1 — Simulation Case Template.docxAgenda.docDebriefing Guide.docFaculty Training PowerPoint.pptxHospital Tech.docxMedication List.docxPrebrief Information.docxMedication Administration Record.docxFaculty Assessment Tool.xlsxStudent Questionnaire.docx [file mep_2374-8265.11054-s001.zip › A. Simulation Case Template.docx]

| **Appendix A: MedEdPORTAL Simulation Case Template**  **SIMULATION CASE TITLE: Using a Resuscitation-Based Simulation Activity to Create an Interprofessional Education Activity for Medical, Nursing, and Pharmacy Students**  AUTHORS: M. Tyson Pillow, M.D., M.Ed., Rebecca Aulbach, Ph.D., Cathy Hatfield, PharmD, Rita DelStritto,PhD, Peggy Landrum, Ph.D., Suzanne Scheller, MS Joel Purkiss, Ph.D., Anne Gill, DrPH  **LEARNER AUDIENCE:** Medical students, nursing students, and pharmacy students | |
| --- | --- |
| **PATIENT NAME: Mr. Smith**  **PATIENT AGE: 32**  **CHIEF COMPLAINT: Shortness of breath/Asthma Exacerbation**  **PHYSICAL SETTING: Inpatient floor with telemetry** | |
|  | |
| **Brief narrative description of case** | The student team will be cross-covering (meaning they are taking care of patients from other teams who are signed out to them overnight) on a patient who has been admitted for an asthma exacerbation/shortness of breath. After getting meds, the patient didn’t really get better. The patient was admitted but feels worse. The patient has asked a nurse to call the medical team to see him. Due to the level of shortness of breath, he can only answer 2-3 words at a time.  The goals of the case are to simulate the care of a critical patient in a resuscitation-based scenario to assess Interprofessional Education Collaborative (IPEC) competencies. Students are put into a case where their individual and team actions affect the outcome, making this activity much more immersive and “real.” |
| **Primary Learning Objectives** | By the end of this activity, learners will be able to:   1. Demonstrate respect for the unique cultures, values, roles/responsibilities, and expertise of other health professions 2. Communicate with team members to clarify each member’s responsibility in executing components of a treatment plan or public health intervention. 3. Use respectful language appropriate for a given difficult situation, crucial conversation, or interprofessional conflict. 4. Reflect on individual and team performance for opportunities to improve. |
| **Critical Actions** | Critical actions of this case include:   - Forming a differential diagnosis - Implementing a dynamic action plan for care of the patient - Placing the patient on nebulized albuterol and atrovent - Administering steroids to the patient - Identifying and avoiding the magnesium sulfate allergy - Providing bag valve mask respirations when the patient decompensates |
| **Learner Preparation or Prework** | No additional learner preparation or prework is assigned for this activity. Students may have already completed the Institute for Healthcare Improvement module, Patient Safety 104 Teamwork and Communication in prior courses. While helpful, the module is not required for the activity. The module is free to anyone with an email address ending in “edu.” and can accessed at: http://app.ihi.org/lmsspa/#/6cb1c614-884b-43ef-9abd-d90849f183d4/3e37eb4a-4928-4d8b-976e-3a2a1a5f2c08 |

| Initial Presentation | | | |
| --- | --- | --- | --- |
| **Initial vital signs** | Starting condition: BP 130/88, respiratory rate 30, speaking 2-3 word sentences, pulse 120, oxygen saturation (O2 sat) 98% | | |
| **Overall Setting and Appearance** | The learners enter the hospital room scenario and find the patient (mannequin) tripoding in acute respiratory distress. The patient is able to communicate in short 2-3 words sentences initially. The patient’s monitors are alarming due to tachycardia and tachypnea. The mannequin has audible wheezing throughout the initial assessment. | | |
| **Confederates (e.g., standardized participants) and their roles in the room at case start** | The case manager (faculty or staff) will act as the patient, respond to requests for labs and studies, and manage the patient monitor. The case manager has no specific verbal prompts, but will answer questions according to the history and physical exam provided in the case materials.  The hospital technician (HT) or facilitator will be available to help students navigate skills and tasks to keep the case flowing (such as assembling nebulizers, drawing up meds, etc…). The HT will be knowledgeable of the case, and will integrate with the team during pre-brief, activity performance, and debrief. The HT will NOT help the team progress in terms of medical knowledge or suggestions for therapies. The HT will stop the team from doing harmful treatments except for administering magnesium sulfate. | | |
| **HPI** | The patient is a 32 y/o male with a history of hypertension (HTN) (managed with medications) and asthma since he was 5 years old. He has had 1-2 “bad” exacerbations per year, but has never been intubated. He has been admitted to an intensive care unit about 3 years ago. He presented to the Emergency Department for shortness of breath, which has been occurring for the last 3-4 days. This started as his normal asthma exacerbation, but he then thought something else was wrong because his inhaler stopped working as well.  Review of Symptoms: increased shortness of breath due to an asthma exacerbation; otherwise negative. Of note, he has had no weight changes, no headaches, or any other issues. | | |
| **Past Medical/Surgical History** | **Medications** | **Allergies** | **Family History** |
| Previous Medical History asthma, HTN  Previous Surgical History none  Soc Hx: occ alcohol no drugs, no smoking | Meds Prior to admission: albuterol, fluticasone/salmeterol inhaler (Diskus) ran out last month, hydrochlorothiazide (HCTZ) | Allergies: Contrast (makes his throat itch) – this allergy should not be remembered by the patient unless directly asked | Hypertension, diabetes mellitus and heart disease run in patient’s family |
| **Physical Examination** | | | |
| **General** | Patient is only able to get out short sentences initially. As the case progresses, patient will only be able to use 1 word sentences. Eventually, patient will stop breathing on their own. | | |
| **HEENT** | Oropharynx clear; dry mucus membranes | | |
| **Neck** | No jugular vein distention trachea midline | | |
| **Lungs** | Inspiratory and expiratory wheezing throughout | | |
| **Cardiovascular** | Tachycardic but no murmurs/gallops/ or rubs | | |
| **Abdomen** | Soft, Non tender, non-distended | | |
| **Neurological** | Awake and alert x3 nonfocal exam | | |
| **Skin** | Diaphoretic | | |
| **GU** | Deferred | | |
| **Psychiatric** | Denies Suicidal ideation/homicidal ideation Auditory verbal hallucinations | | |

| Instructor Notes - Changes and CASE Branch Points  This section should be a list with detailed description of each step than may happen during the case. If medications are given, what is the response? Do changes occur at certain time points? Should the nurse or other participant prompt the learners at given points? Should new actors or participants enter, and when? Are there specific things the patient will say or do at given times? There are a few examples given, but it is expected that most cases will have many more changes and potential branch points.  If you have a more complex branching algorithm than can be accommodated by the structure below, feel free to replace this section with your own. Look at some recent simulation publications on MedEdPORTAL for examples. | | |
| --- | --- | --- |
| **Intervention / Time point** | **Change in Case** | **Additional Information** |
| No intervention within the first 5 minutes of starting the case. | Patient begins to deteriorate**.** Patient progresses to state “worsened condition 1.” | Any request for imaging, labs, placement of oxygen, or administration of medications will count as an intervention. |
| Albuterol AND ipratropium nebs are given. | Patient condition remains at “starting state” for an additional 60-90 seconds (see below). | Both medications MUST be given to stabilize the patient the current state. |
| Steroids are administered via IV route. | Patient condition remains at “starting state” for an additional 60-90 seconds (see below). | Oral administration of steroids is not preferred, but will not be penalized. |
| Team requests CPAP or BiPAP for the patient | No change | CPAP/BiPAP not available, but recognizing the indication in this case will be noted in the debriefing. |
| Epinephrine is administered via IM route | If the patient was in state “worsened condition 1,” then they improve to “starting state.” | Dosing may be incorrect, but the team will not be penalized. Epinephrine given after 10 min into the case will not improve the patient condition. |
| Epinephrine is administered via IV route | If the patient was in state “worsened condition 1,” then they improve to “starting state.” | Hospital tech will advise team to give the epinephrine IM instead of IV. If the team continues with the IV dosing despite prompting, they will not be penalized, but will be discussed in the debriefing. |
| Magnesium sulfate is administered to the patient | The patient complains of feeling itchy and skin becomes red. No change in vital signs. | The team should recognize the allergic reaction and give diphenhydramine. If the team does not give diphenhydramine, the patient remains itchy and red but the cardiovascular state does not change. |
| 7 minutes into the case | Patient deteriorates to “worsened condition 1” if not already in this state. This change in condition can be delayed by 60-90 seconds for each of the interventions noted above with benefit to the patient for a total of 3 min increased duration. | The patient will deteriorate to “worsened condition 1” if not already in that state between 7-10 minutes in the case. |
| 12 minutes into the case | Patient condition deteriorates to “worsened condition #2” despite previous care received. Patient becomes apneic. | Team should recognize respiratory arrest, but patient still has a pulse. |
| Bag-valve mask (BVM) ventilations are initiated. | O2 sat becomes readable at 95%. | Respirations are supported with BVM. The team may request anesthesia to intubate or may attempt intubation themselves. |

States

- Starting condition: BP 130/88, RR30, speaking 2-3 word sentences, tripoding, pulse 120, sats 98%
- Worse condition #1: 140/90, RR50, speaking 1 word sentences, tripoding, pulse 150, sats 90%
- Worse condition #2: BP 110/70, RR0, not able to speak, unconscious but maintaining a pulse, sats unreadable (until team starts to use BVM for the patient)

**Ideal Scenario Flow**

Provide a detailed narrative description of the way this case should flow if participants perform in the ideal fashion.

The team enters the rooms and quickly assesses the patient’s ABCs. With ABC’s intact, the team begins to form a differential diagnosis while treating for worsening asthma exacerbation. A focused history is taken while interventions are administered. Albuterol and ipratropium nebs are given, as well as IV steroids. IM epinephrine is also administered. The team may also request CPAP/BiPAP, but it is not available. The patient’s allergy to magnesium sulfate should be identified in the history or on the medication administration record and the medication held. The team may ask for a bedside chest x-ray, but the radiology tech does not arrive during the case. The patient’s condition continues to slowly deteriorate despite optimal treatment and eventually, the patient goes into respiratory arrest (but maintains a pulse). The team should then support respirations with BVM (and possibly oral/nasal airway placement) and either request anesthesia support or attempt intubation themselves. The case ends once ventilations are adequately supported and the decision to intubate has been made.

**Anticipated Management Mistakes**

Provide a list of management errors or difficulties that are commonly encountered when using this simulation case.

1. Lack of knowledge at nebulizer assembly/medication administration: Teams sometimes need help with assembling the nebulizer or administering medications via the IV line. The hospital technician is commonly utilized to help navigate this problem.
2. Failure to consider magnesium sulfate treatment: Some teams fail to even consider magnesium sulfate treatment rather than recognizing the allergy and avoiding the therapy for that reason. This issue is addressed in the debriefing.
3. Individual or team knowledge deficits or professionalism issues: Infrequently, serious issues arise during the case on either the individual or team level. Faculty observers and assessors may use “yellow cards” to write the issue down but allow the case to continue. After debriefing, the case manage collects the cards and distributes to discipline specific faculty to address the issue with the student before they leave.
