# Supplementary material for: Using a Resuscitation-Based Simulation Activity to Create an Interprofessional Education Activity for Medical, Nursing, and Pharmacy Students
Source: MedEdPORTAL. 2020 Dec 11;16:11054. doi: 10.15766/mep_2374-8265.11054 (PMC7732132; doi:10.15766/mep_2374-8265.11054)
Supplement: Supplementary file 1 — Simulation Case Template.docxAgenda.docDebriefing Guide.docFaculty Training PowerPoint.pptxHospital Tech.docxMedication List.docxPrebrief Information.docxMedication Administration Record.docxFaculty Assessment Tool.xlsxStudent Questionnaire.docx [file mep_2374-8265.11054-s001.zip › D. Faculty Training PowerPoint.pptx]

## Slide 1
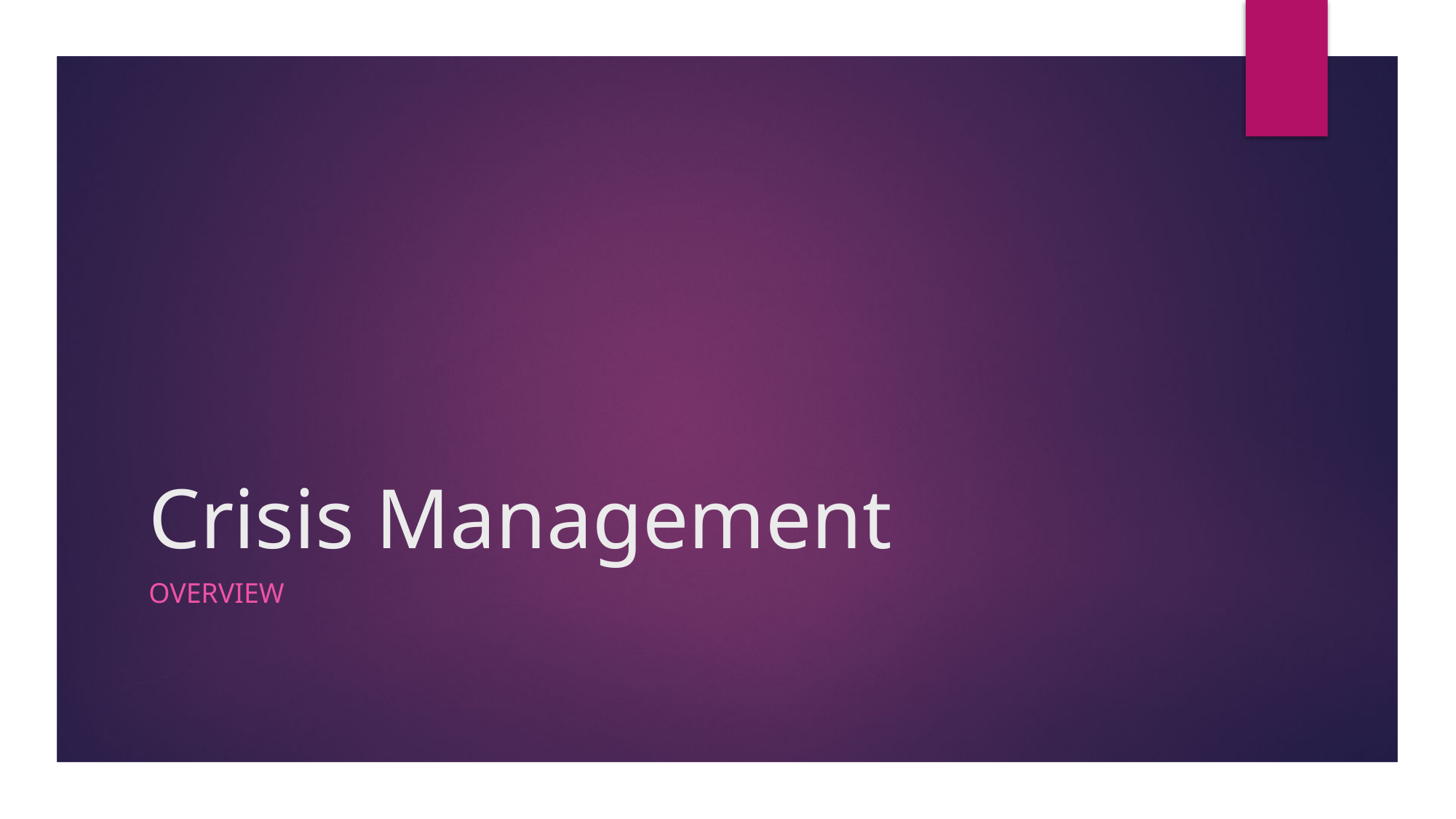

# Crisis Management
Overview

## Slide 2
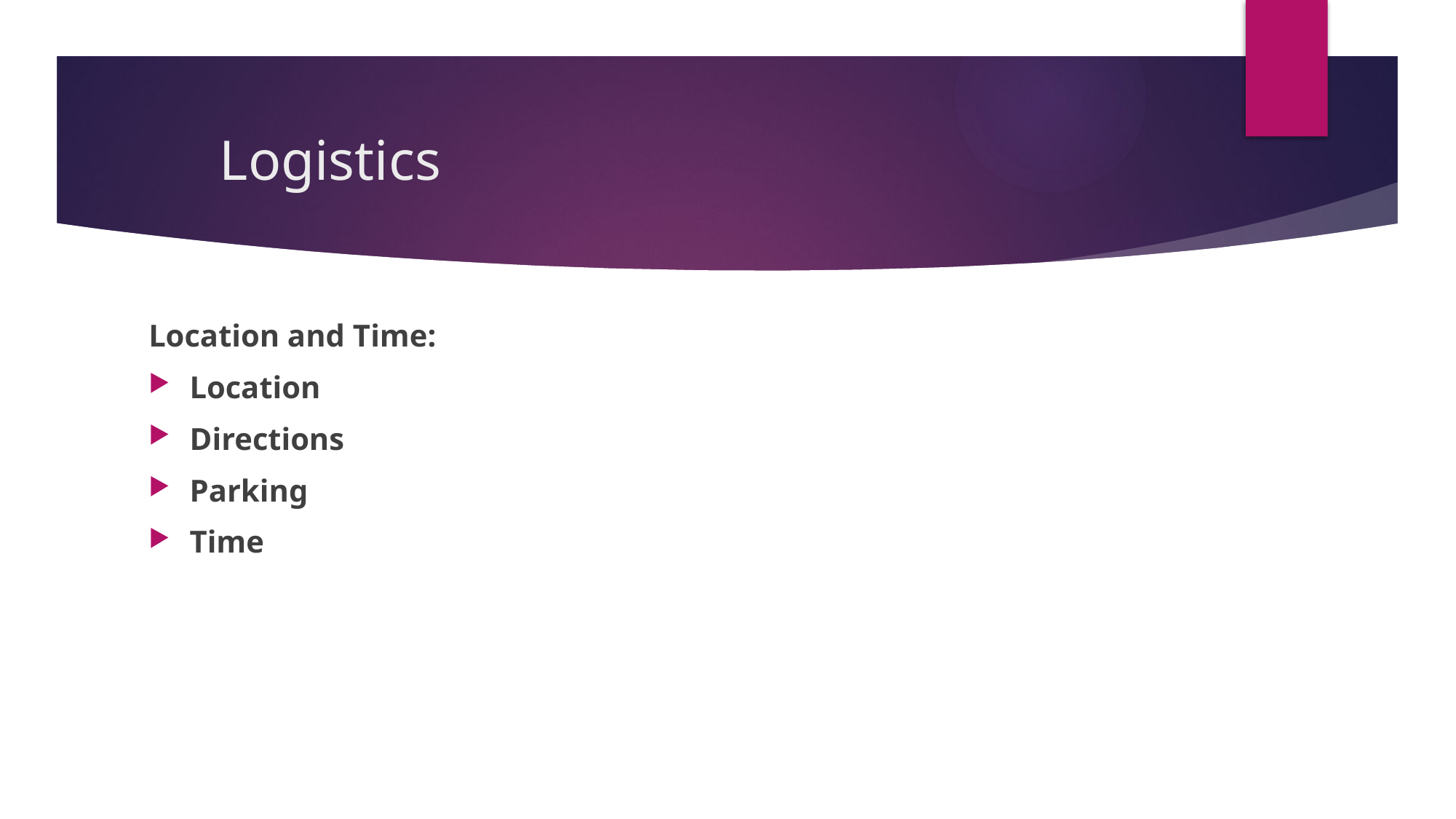

# Logistics
Location and Time:
Location
Directions
Parking
Time

## Slide 3
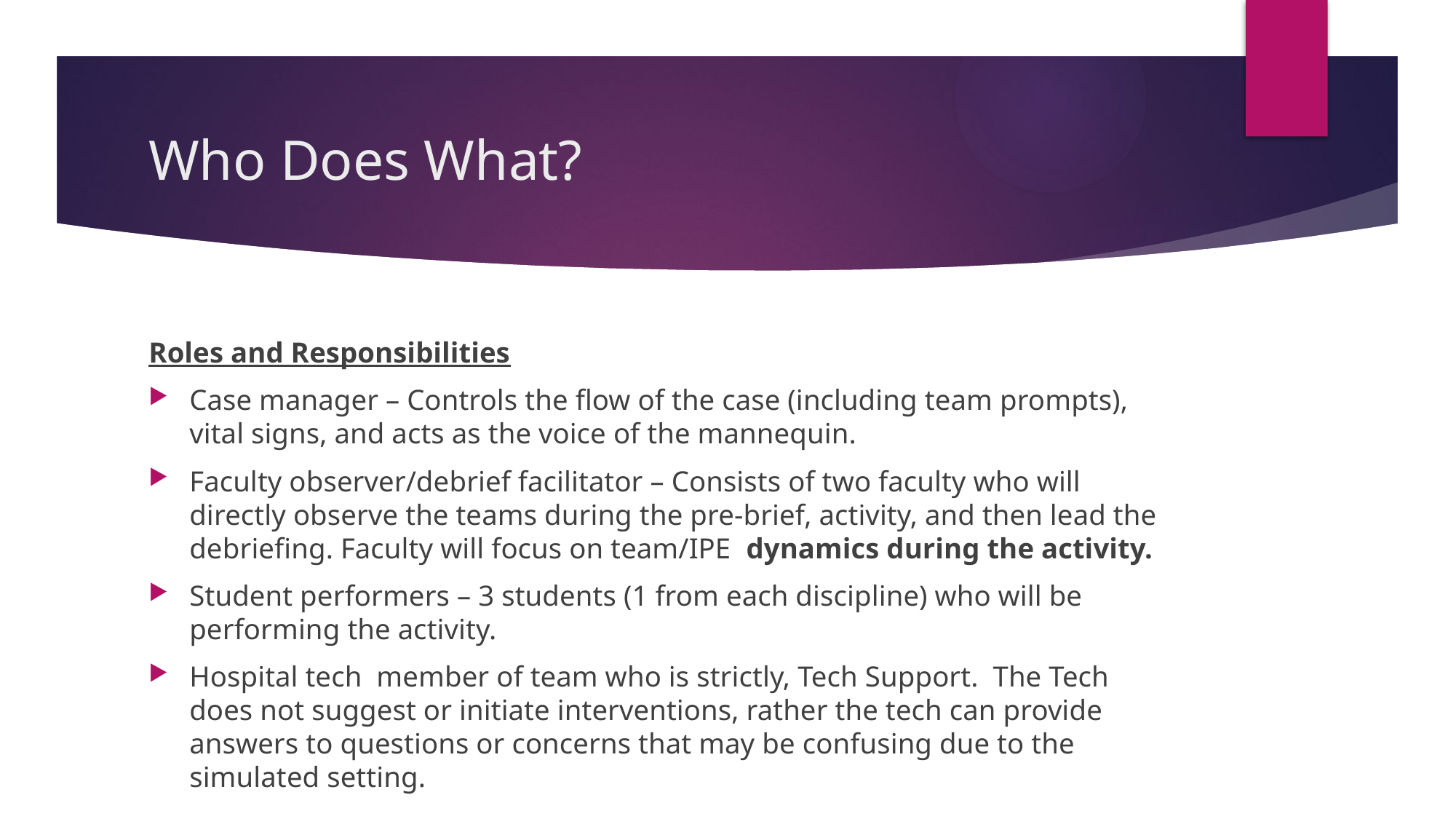

# Who Does What?
Roles and Responsibilities
Case manager – Controls the flow of the case (including team prompts), vital signs, and acts as the voice of the mannequin.
Faculty observer/debrief facilitator – Consists of two faculty who will directly observe the teams during the pre-brief, activity, and then lead the debriefing. Faculty will focus on team/IPE dynamics during the activity.
Student performers – 3 students (1 from each discipline) who will be performing the activity.
Hospital tech member of team who is strictly, Tech Support. The Tech does not suggest or initiate interventions, rather the tech can provide answers to questions or concerns that may be confusing due to the simulated setting.

## Slide 4
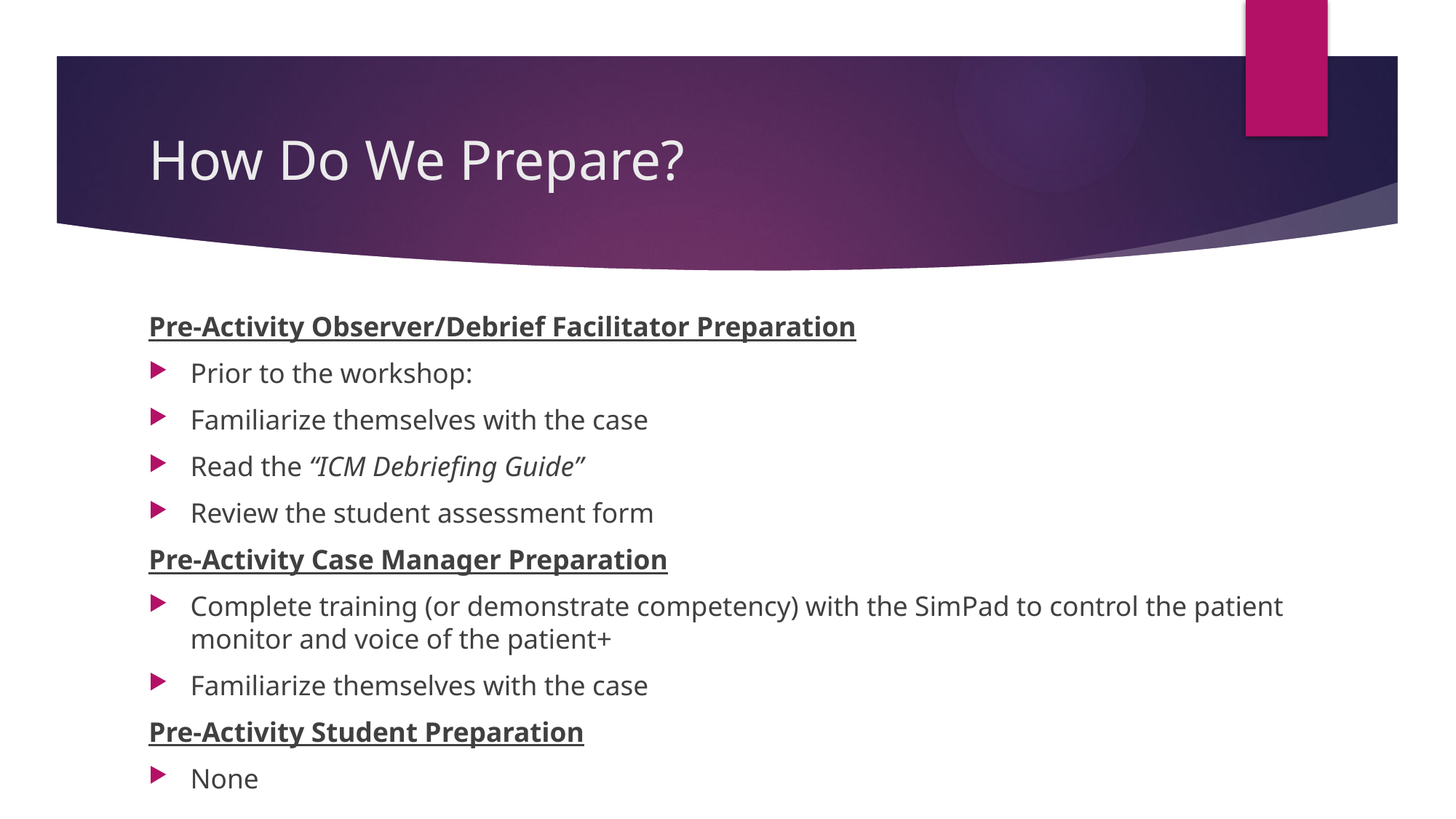

# How Do We Prepare?
Pre-Activity Observer/Debrief Facilitator Preparation
Prior to the workshop:
Familiarize themselves with the case
Read the “ICM Debriefing Guide”
Review the student assessment form
Pre-Activity Case Manager Preparation
Complete training (or demonstrate competency) with the SimPad to control the patient monitor and voice of the patient+
Familiarize themselves with the case
Pre-Activity Student Preparation
None

## Slide 5
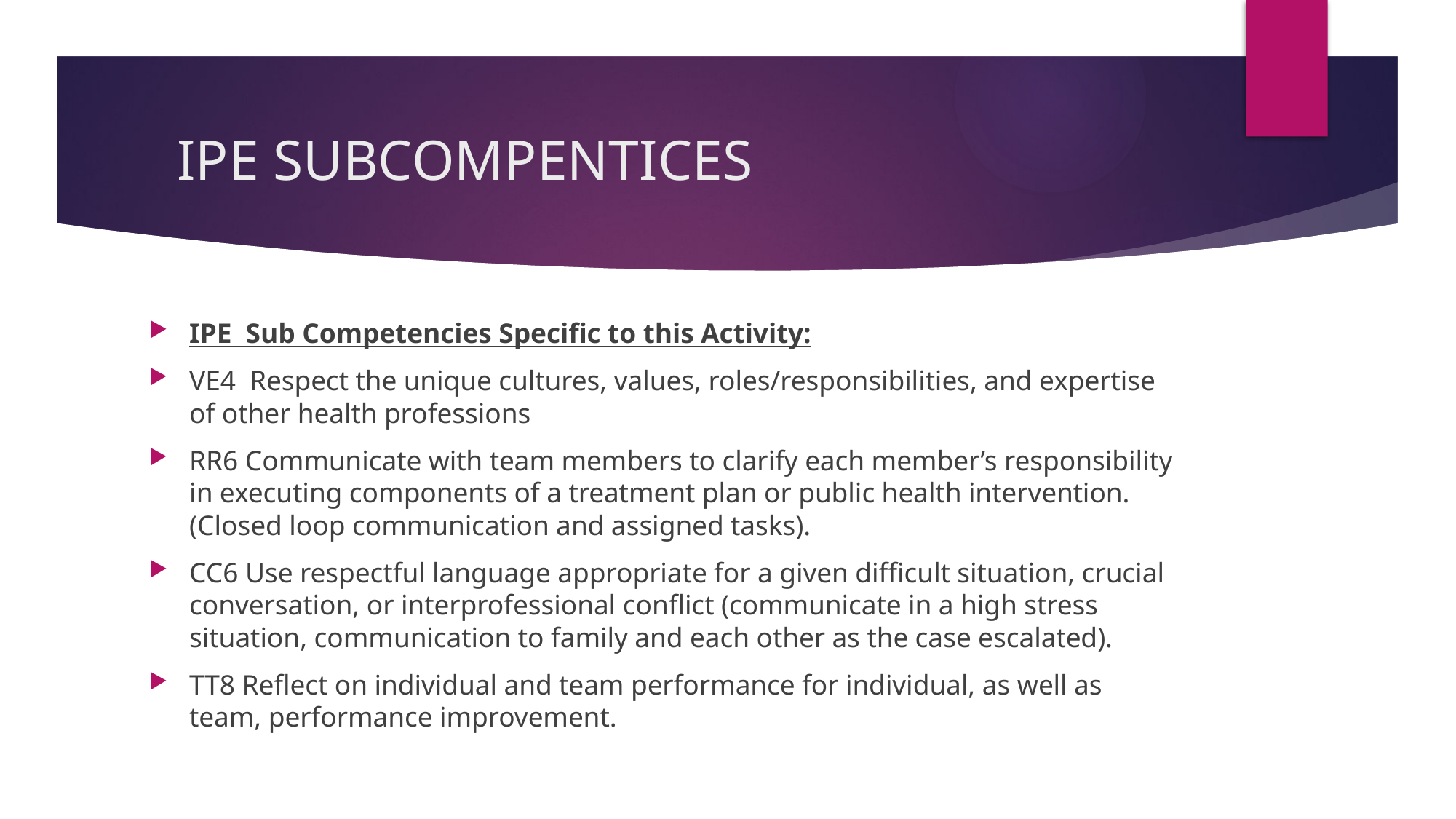

# IPE SUBCOMPENTICES
IPE Sub Competencies Specific to this Activity:
VE4 Respect the unique cultures, values, roles/responsibilities, and expertise of other health professions
RR6 Communicate with team members to clarify each member’s responsibility in executing components of a treatment plan or public health intervention. (Closed loop communication and assigned tasks).
CC6 Use respectful language appropriate for a given difficult situation, crucial conversation, or interprofessional conflict (communicate in a high stress situation, communication to family and each other as the case escalated).
TT8 Reflect on individual and team performance for individual, as well as team, performance improvement.

## Slide 6
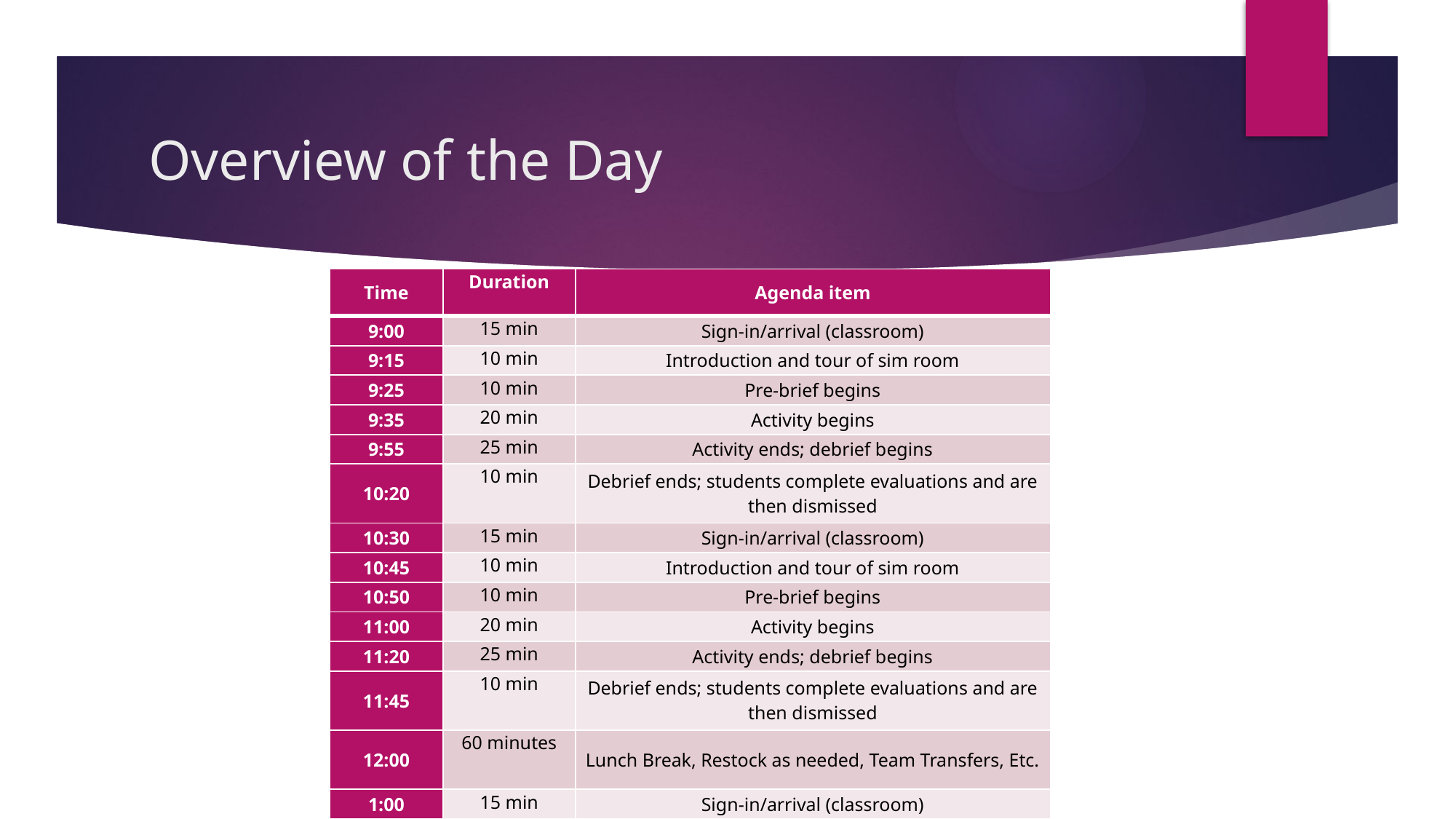

# Overview of the Day
| Time | Duration | Agenda item |
| --- | --- | --- |
| 9:00 | 15 min | Sign-in/arrival (classroom) |
| 9:15 | 10 min | Introduction and tour of sim room |
| 9:25 | 10 min | Pre-brief begins |
| 9:35 | 20 min | Activity begins |
| 9:55 | 25 min | Activity ends; debrief begins |
| 10:20 | 10 min | Debrief ends; students complete evaluations and are then dismissed |
| 10:30 | 15 min | Sign-in/arrival (classroom) |
| 10:45 | 10 min | Introduction and tour of sim room |
| 10:50 | 10 min | Pre-brief begins |
| 11:00 | 20 min | Activity begins |
| 11:20 | 25 min | Activity ends; debrief begins |
| 11:45 | 10 min | Debrief ends; students complete evaluations and are then dismissed |
| 12:00 | 60 minutes | Lunch Break, Restock as needed, Team Transfers, Etc. |
| 1:00 | 15 min | Sign-in/arrival (classroom) |

## Slide 7
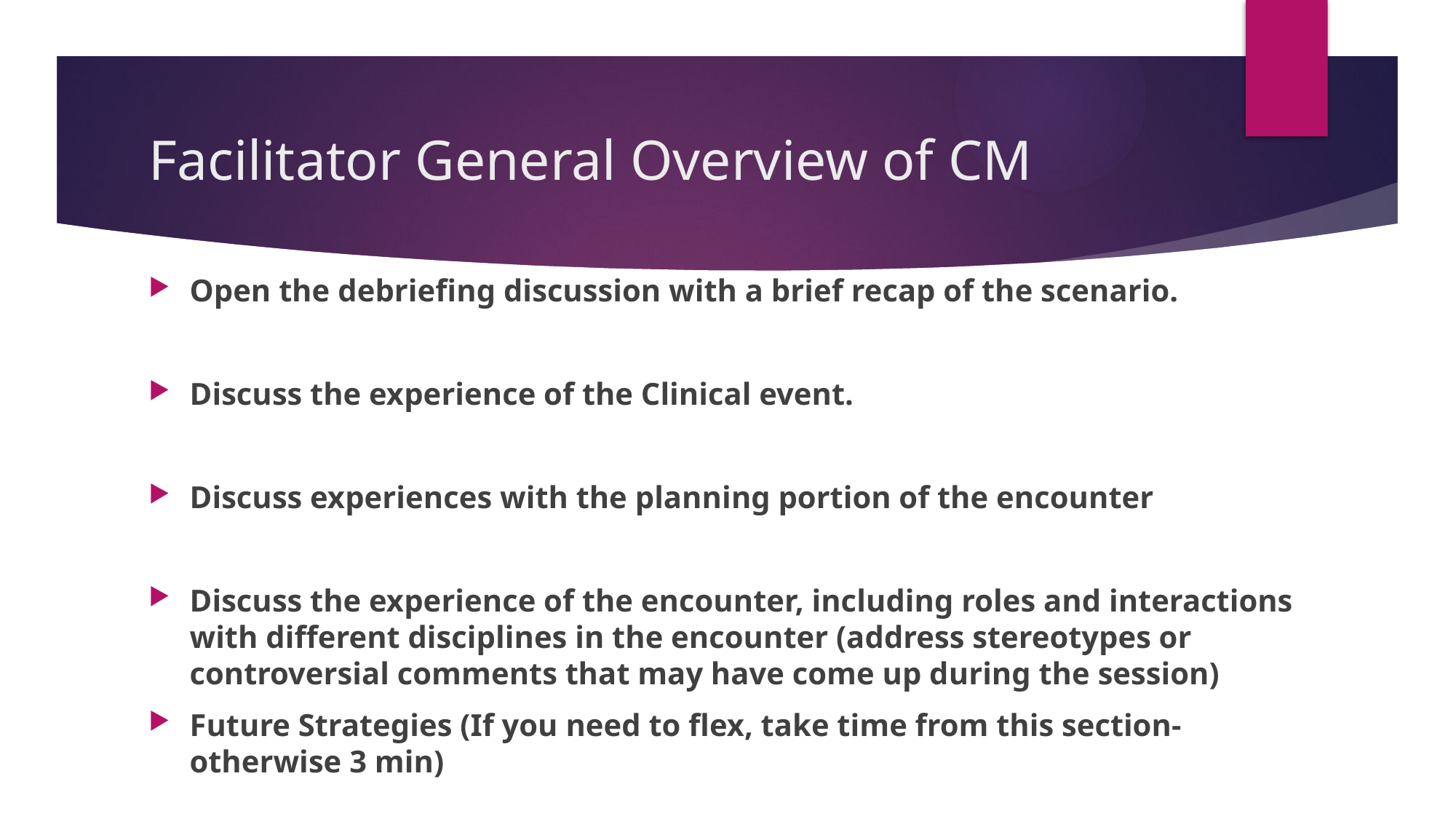

# Facilitator General Overview of CM
Open the debriefing discussion with a brief recap of the scenario.
Discuss the experience of the Clinical event.
Discuss experiences with the planning portion of the encounter
Discuss the experience of the encounter, including roles and interactions with different disciplines in the encounter (address stereotypes or controversial comments that may have come up during the session)
Future Strategies (If you need to flex, take time from this section-otherwise 3 min)

## Slide 8
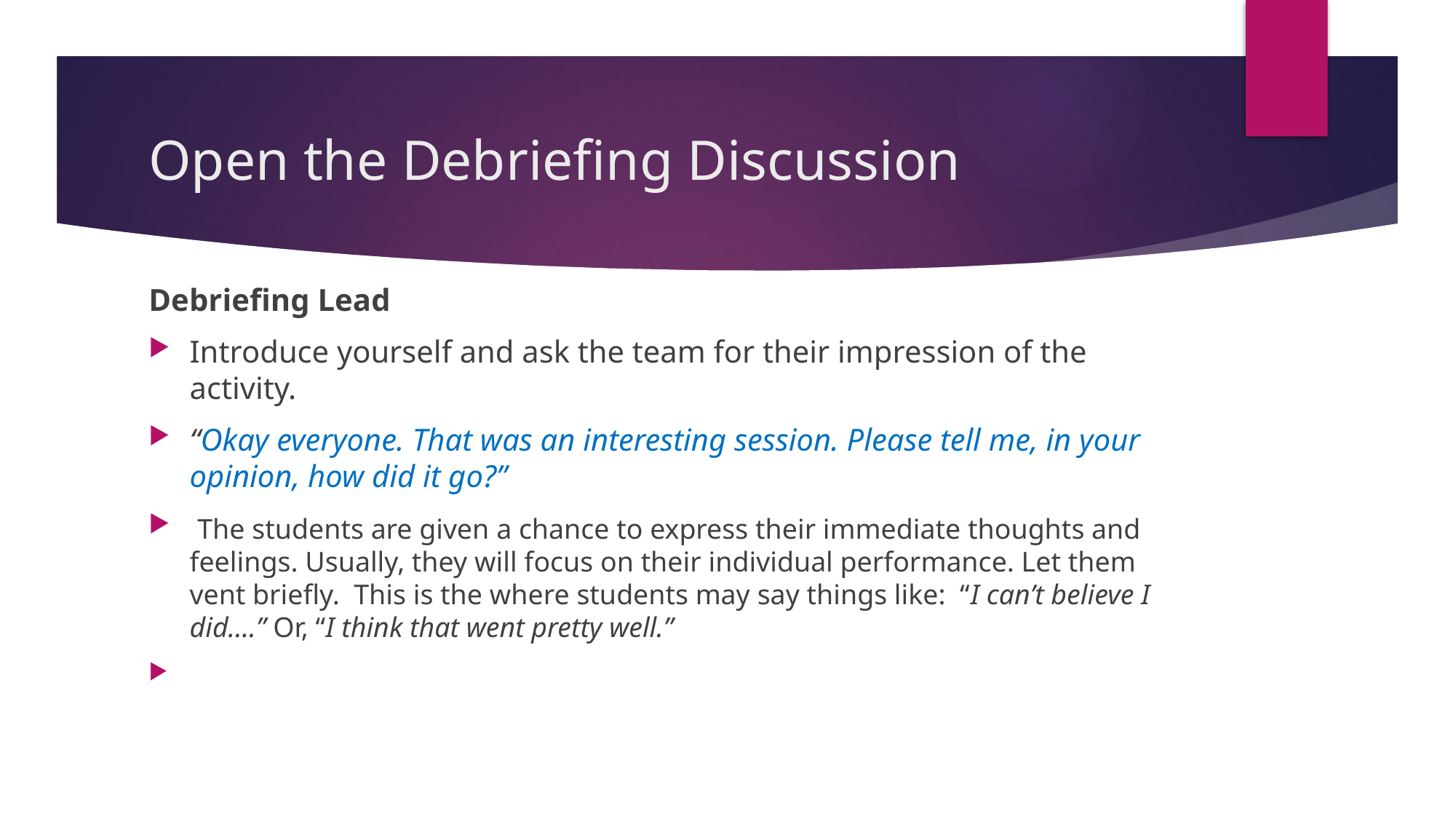

# Open the Debriefing Discussion
Debriefing Lead
Introduce yourself and ask the team for their impression of the activity.
“Okay everyone. That was an interesting session. Please tell me, in your opinion, how did it go?”
 The students are given a chance to express their immediate thoughts and feelings. Usually, they will focus on their individual performance. Let them vent briefly. This is the where students may say things like: “I can’t believe I did….” Or, “I think that went pretty well.”

## Slide 9
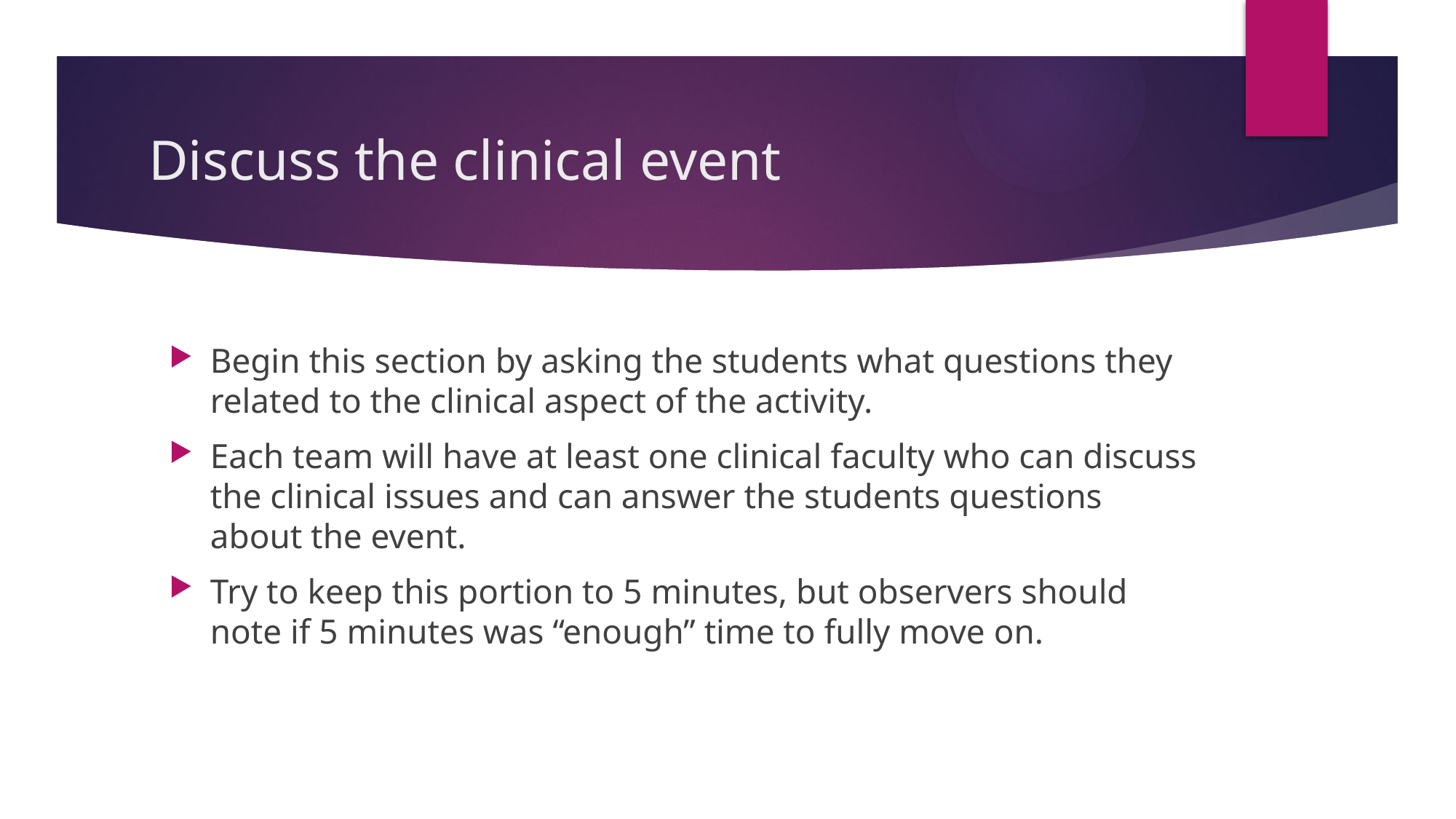

# Discuss the clinical event
Begin this section by asking the students what questions they related to the clinical aspect of the activity.
Each team will have at least one clinical faculty who can discuss the clinical issues and can answer the students questions about the event.
Try to keep this portion to 5 minutes, but observers should note if 5 minutes was “enough” time to fully move on.

## Slide 10
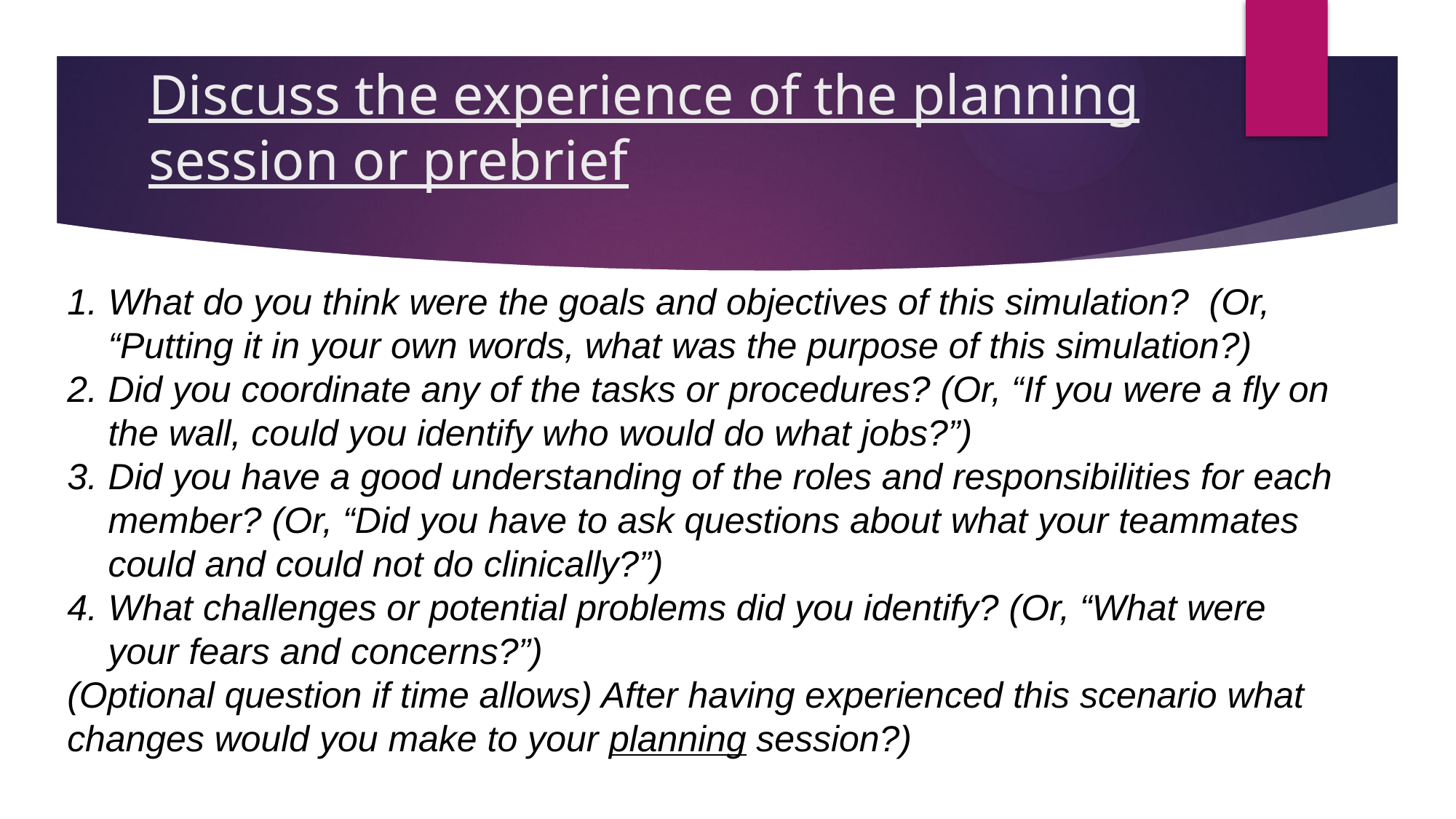

# Discuss the experience of the planning session or prebrief
What do you think were the goals and objectives of this simulation? (Or, “Putting it in your own words, what was the purpose of this simulation?)
Did you coordinate any of the tasks or procedures? (Or, “If you were a fly on the wall, could you identify who would do what jobs?”)
Did you have a good understanding of the roles and responsibilities for each member? (Or, “Did you have to ask questions about what your teammates could and could not do clinically?”)
What challenges or potential problems did you identify? (Or, “What were your fears and concerns?”)
(Optional question if time allows) After having experienced this scenario what changes would you make to your planning session?)

## Slide 11
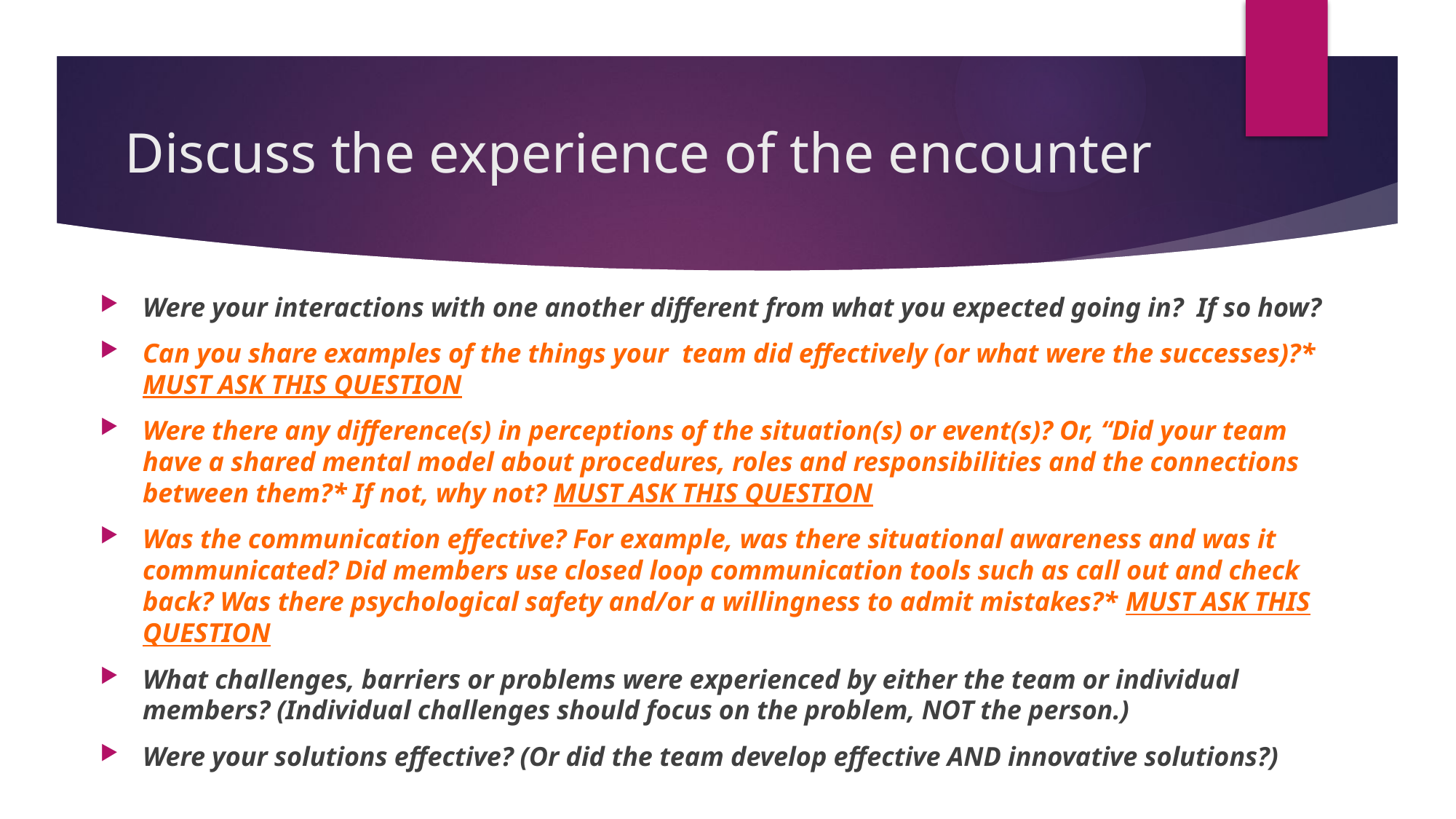

# Discuss the experience of the encounter
Were your interactions with one another different from what you expected going in? If so how?
Can you share examples of the things your team did effectively (or what were the successes)?* MUST ASK THIS QUESTION
Were there any difference(s) in perceptions of the situation(s) or event(s)? Or, “Did your team have a shared mental model about procedures, roles and responsibilities and the connections between them?* If not, why not? MUST ASK THIS QUESTION
Was the communication effective? For example, was there situational awareness and was it communicated? Did members use closed loop communication tools such as call out and check back? Was there psychological safety and/or a willingness to admit mistakes?* MUST ASK THIS QUESTION
What challenges, barriers or problems were experienced by either the team or individual members? (Individual challenges should focus on the problem, NOT the person.)
Were your solutions effective? (Or did the team develop effective AND innovative solutions?)

## Slide 12
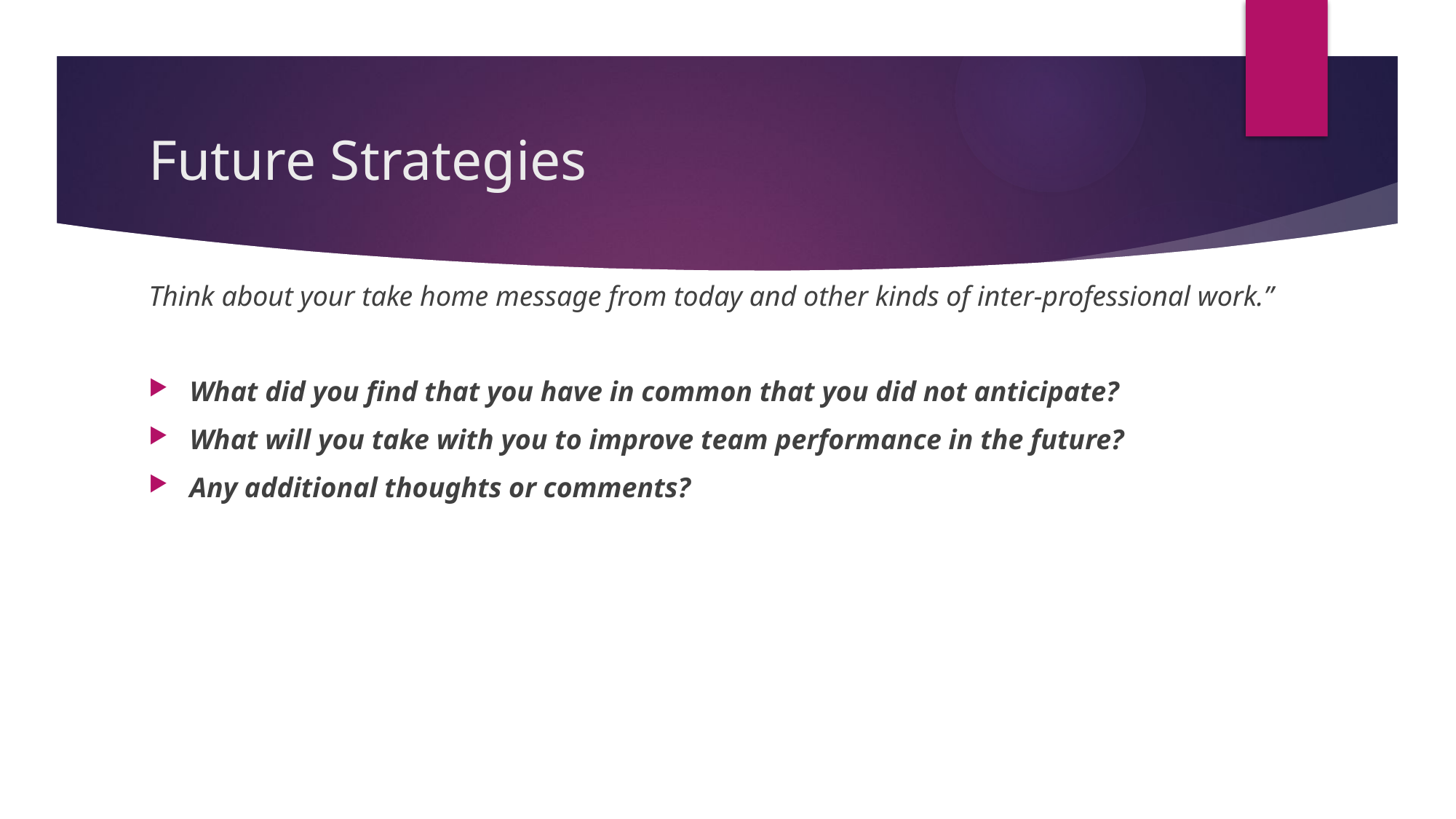

# Future Strategies
Think about your take home message from today and other kinds of inter-professional work.”
What did you find that you have in common that you did not anticipate?
What will you take with you to improve team performance in the future?
Any additional thoughts or comments?

## Slide 13
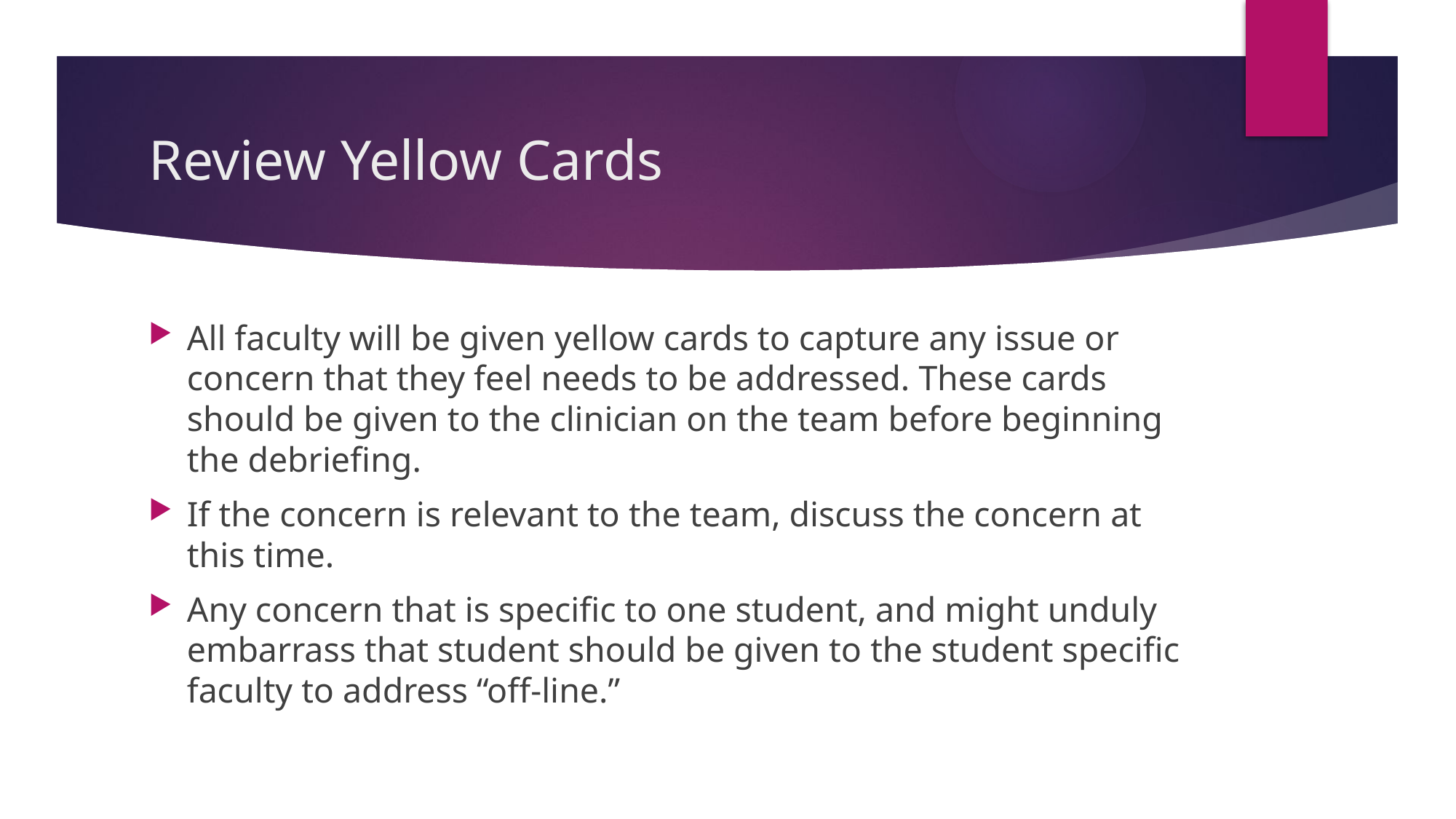

# Review Yellow Cards
All faculty will be given yellow cards to capture any issue or concern that they feel needs to be addressed. These cards should be given to the clinician on the team before beginning the debriefing.
If the concern is relevant to the team, discuss the concern at this time.
Any concern that is specific to one student, and might unduly embarrass that student should be given to the student specific faculty to address “off-line.”

## Slide 14
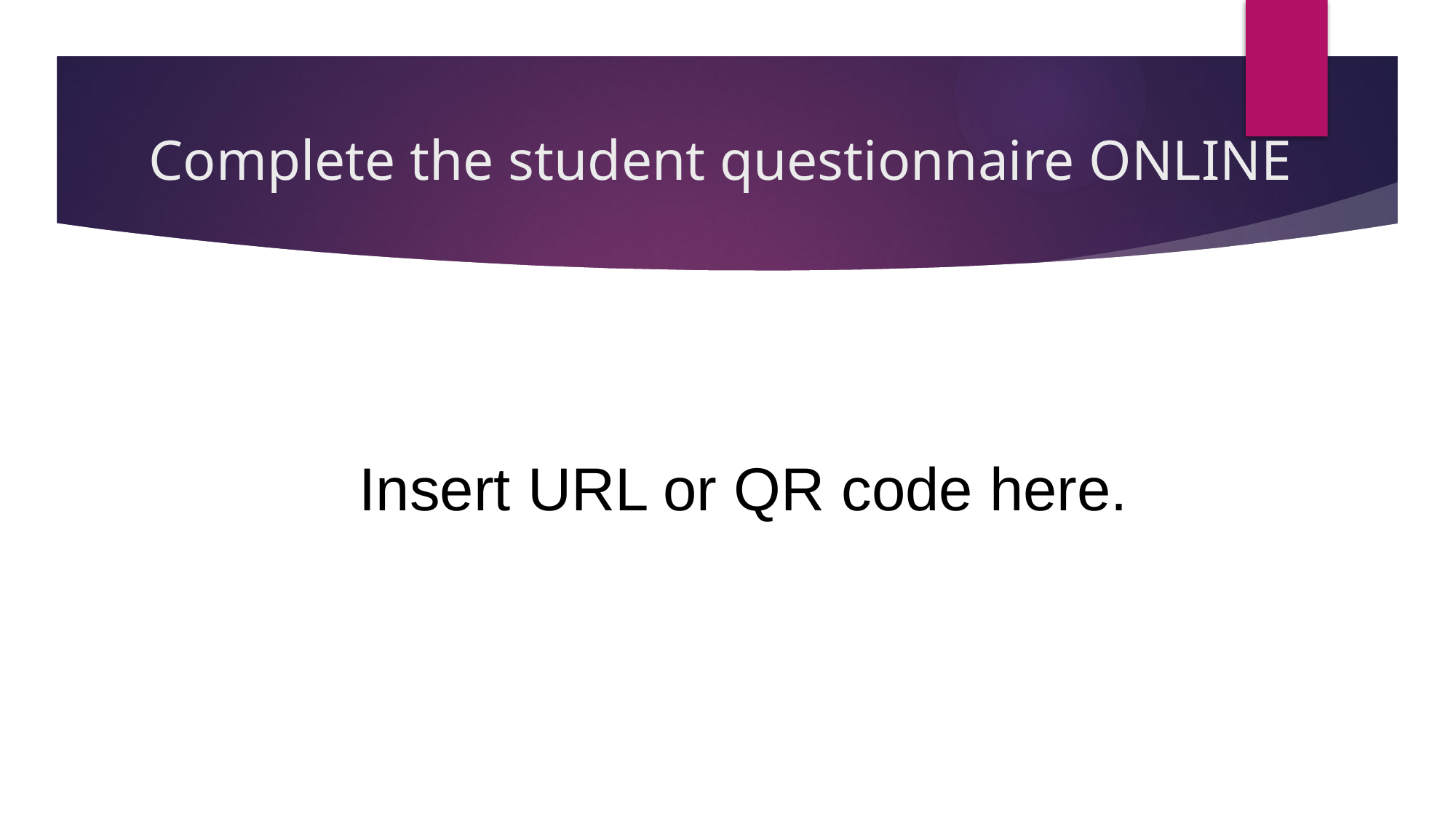

# Complete the student questionnaire ONLINE
Insert URL or QR code here.

## Slide 15
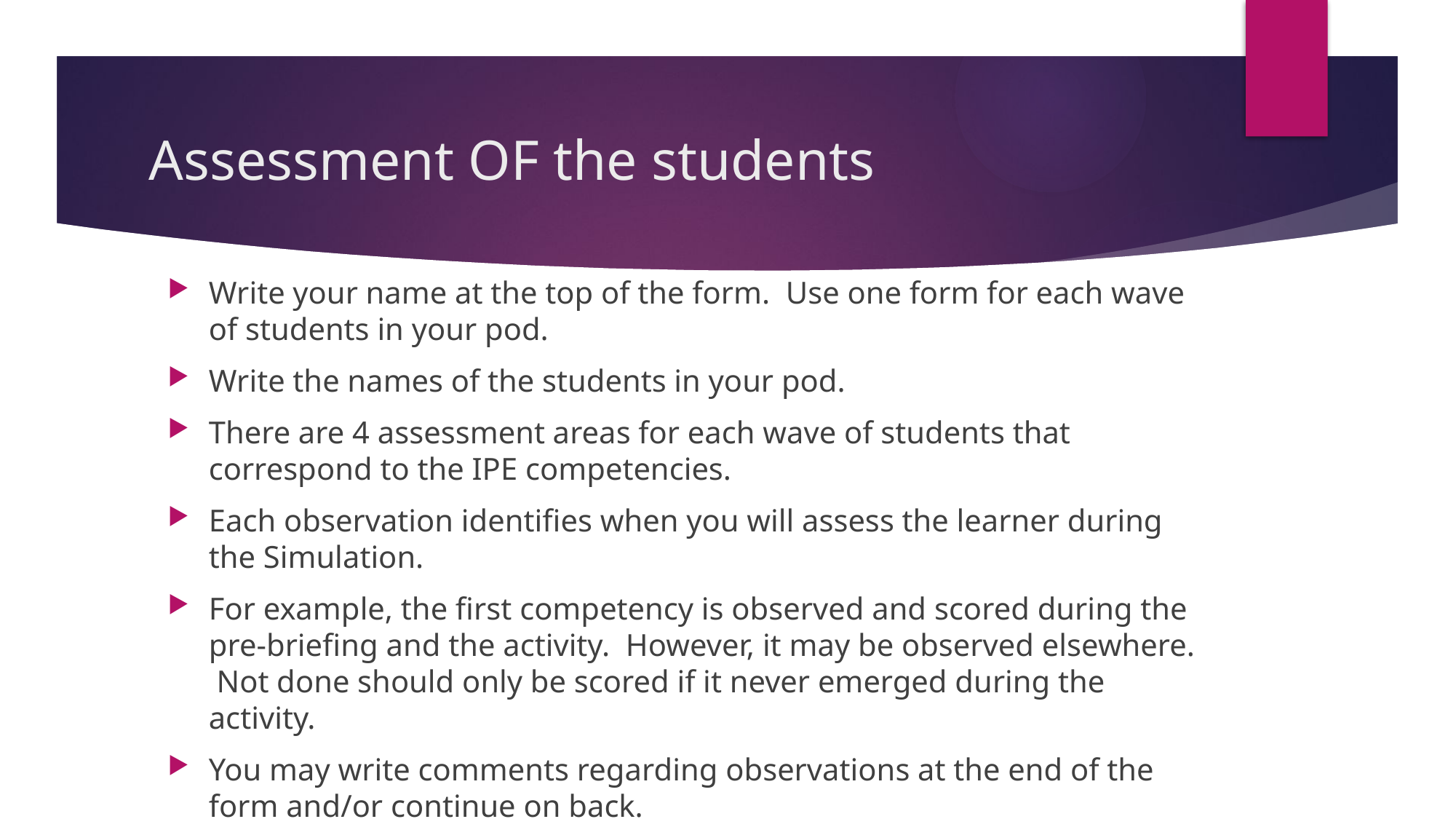

# Assessment OF the students
Write your name at the top of the form. Use one form for each wave of students in your pod.
Write the names of the students in your pod.
There are 4 assessment areas for each wave of students that correspond to the IPE competencies.
Each observation identifies when you will assess the learner during the Simulation.
For example, the first competency is observed and scored during the pre-briefing and the activity. However, it may be observed elsewhere. Not done should only be scored if it never emerged during the activity.
You may write comments regarding observations at the end of the form and/or continue on back.

## Slide 16
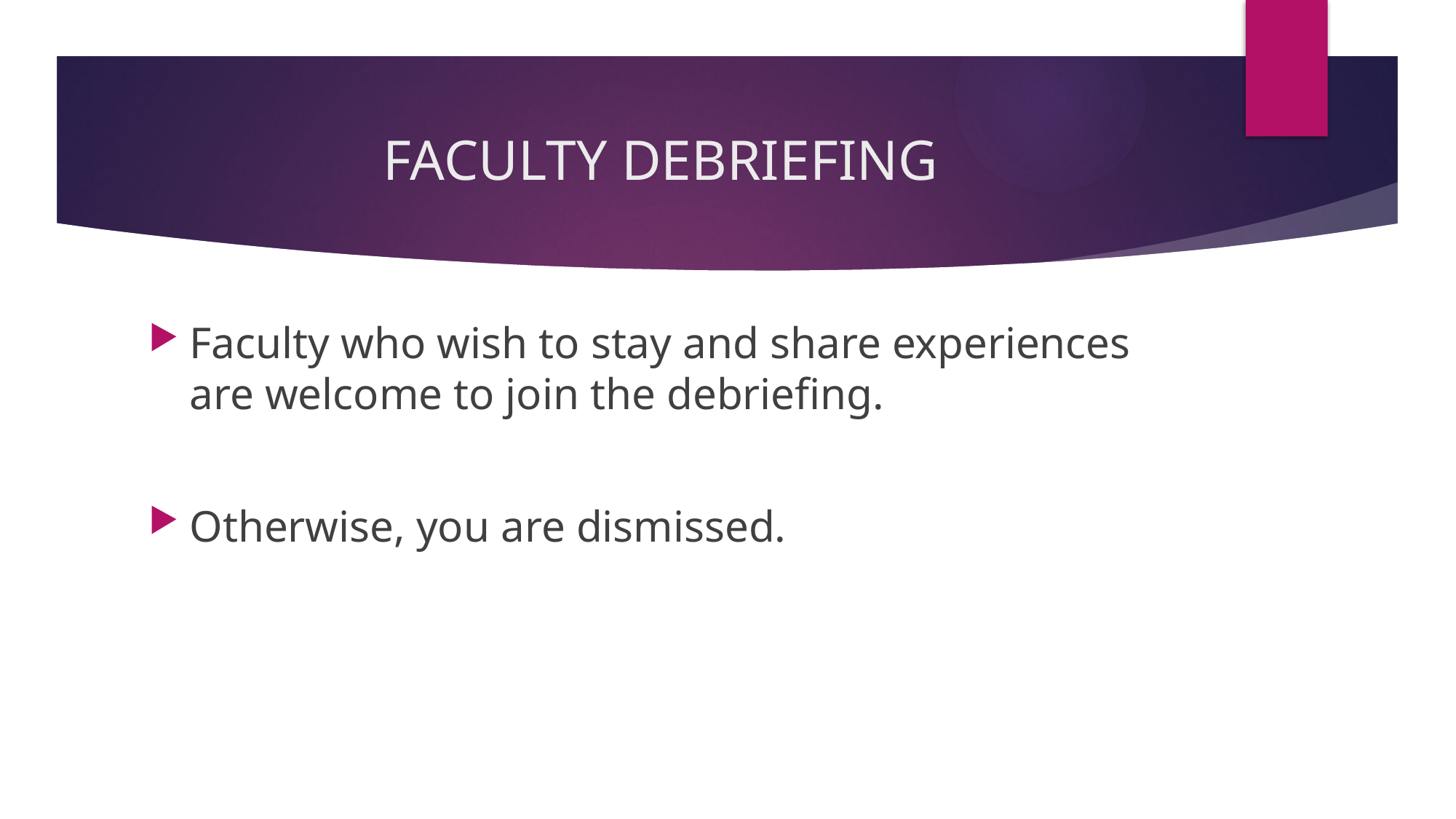

# FACULTY DEBRIEFING
Faculty who wish to stay and share experiences are welcome to join the debriefing.
Otherwise, you are dismissed.
