# Supplementary material for: Using a Resuscitation-Based Simulation Activity to Create an Interprofessional Education Activity for Medical, Nursing, and Pharmacy Students
Source: MedEdPORTAL. 2020 Dec 11;16:11054. doi: 10.15766/mep_2374-8265.11054 (PMC7732132; doi:10.15766/mep_2374-8265.11054)
Supplement: Supplementary file 1 — Simulation Case Template.docxAgenda.docDebriefing Guide.docFaculty Training PowerPoint.pptxHospital Tech.docxMedication List.docxPrebrief Information.docxMedication Administration Record.docxFaculty Assessment Tool.xlsxStudent Questionnaire.docx [file mep_2374-8265.11054-s001.zip › E. Hospital Tech.docx]

**Role of the Tech**

**Assumptions:** The Tech” is considered a member of the team, however, this role is strictly, “Technical Support.” The “Tech” does not suggest or initiate interventions, rather the tech can provide answers to questions or concerns that may be a confusing due to the simulated setting.

**The Tech will orient the team members to the simulation bay in the 10 minute allotted time. The orientation will include the following:**

**Manikin:**

1. Demonstrate using a stethoscope where learners will hear breath sounds and heart sounds and ask them listen.
2. Ask the case manager to demonstrate the manikin speaking.
3. Direct learners to palpate the pulse on the manikin.
4. Inform learners that they can push meds and intubate if needed.

**Monitor:**

1. Identify the vital signs displayed on the hospital monitor.
2. Identify the heart rate, blood pressure, and O2 sat read outs on the monitor
3. Inform learners that the monitor is calibrated and accurate. Reinforce that the digital readouts are also correct.

**Crash Cart:**

1. What drugs are available, what drugs are not available.
2. How things are organized on the cart
3. Where to find the MAR and allergy information

**General:**

1. Direct learners attention to the O2 set up
2. Direct learners attention to the suction

**Inform learners that:**

- The Tech will perform the role of informing the team of lab results, imaging results and other service concerns upon request of the team.
- The Tech can relay or receive messages to or from the family if requested.

Allow students to explore and touch items in the room up to the 10 minutes allotted for this part of the activity.
