# Supplementary material for: Using a Resuscitation-Based Simulation Activity to Create an Interprofessional Education Activity for Medical, Nursing, and Pharmacy Students
Source: MedEdPORTAL. 2020 Dec 11;16:11054. doi: 10.15766/mep_2374-8265.11054 (PMC7732132; doi:10.15766/mep_2374-8265.11054)
Supplement: Supplementary file 1 — Simulation Case Template.docxAgenda.docDebriefing Guide.docFaculty Training PowerPoint.pptxHospital Tech.docxMedication List.docxPrebrief Information.docxMedication Administration Record.docxFaculty Assessment Tool.xlsxStudent Questionnaire.docx [file mep_2374-8265.11054-s001.zip › F. Medication List.docx]

**Interprofessional Crisis Management**

Medication List

2018

Albuterol sulfate 0.083% 2.5 mg/3 ml)

Ipratropium bromide 0.02% 0.5 mg/2.5 ml

Atropine sulfate 1 mg/ml

Adenosine 3 mg/mL

Calcium chloride 10% 100 mg/ml

Dexamethasone sodium phosphate 4 mg/ml

Diltiazem HCl 25 mg/5 ml

Diphenhydramine HCl 50 mg/ml

Flumazenil 1 mg/10 ml

Vasopressin 20 units/ml

50% Dextrose 25g /50 ml

Amiodarone HCl 150 mg/3 ml

Nitroglycerin 50 mg/10 ml

8.4% Sodium bicarbonate 1 mEq/ml

Dopamine HCl 40 mg/ml

Furosemide 10 mg/ml

50% Magnesium sulfate 4 mEq/ml

Phenylephrine 10 mg/ml

Epinephrine 1:1000 1 mg/ml

Epinephrine 1:10,000 0.1 mg/ml

Naloxone 0.4 mg/ml

Digoxin 0.5 mg/2 ml

Normal saline 1000 ml bag
