# Supplementary material for: Using a Resuscitation-Based Simulation Activity to Create an Interprofessional Education Activity for Medical, Nursing, and Pharmacy Students
Source: MedEdPORTAL. 2020 Dec 11;16:11054. doi: 10.15766/mep_2374-8265.11054 (PMC7732132; doi:10.15766/mep_2374-8265.11054)
Supplement: Supplementary file 1 — Simulation Case Template.docxAgenda.docDebriefing Guide.docFaculty Training PowerPoint.pptxHospital Tech.docxMedication List.docxPrebrief Information.docxMedication Administration Record.docxFaculty Assessment Tool.xlsxStudent Questionnaire.docx [file mep_2374-8265.11054-s001.zip › G. Prebrief Information.docx]

**Interprofessional Crisis Management Activity**

**Pre-Brief Information**

Synopsis

The team is cross-covering on the weekend and has been called to the bedside for a patient from Team E who was admitted for an asthma exacerbation a few hours ago. The patient is complaining about increased shortness of breath. The team is briefly able to review the patient’s chart, which shows the following:

Sign-out note:

32 y/o patient with a history of asthma, hypertension (HTN) who presents complaining of shortness of breath. In the ED, the patient was noted to have significant diffuse wheezing, and given albuterol/ipratropium nebs x 2 with some improvement of symptoms. Pt denied chest pain (CP) or other symptoms, so acute coronary syndrome (ACS) work-up not pursued.

PMHx: Asthma since 5 y/o; HTN (stage 2)

PSHx: none

Allergies: Iodine

Meds PTA: albuterol, fluticasone/salmeterol diskus, hydrochlorothiazide (HCTZ)

FMHx: HTN, DM, and heart disease

Soc Hx: occ EtOH, no drugs, no smoking

CXR (this admission) – slightly increased interstitial markings but otherwise, no acute abnormalities

EKG: sinus tachycardia at rate of 120; no S1, Q3, T3; no other abnormalities

Labs (in the ED):

CBC

WBC 8.6

Hgb 14.2

Plt 345

BMP

Na 138

K 3.2

Cl 100

CO2 23

BUN 20

Cr 1.2

BGL - 110

UA – normal

ABG

7.35/50/70

Cross-cover plan: Continue monitoring and give nebs as needed.
