# Supplementary material for: Using a Resuscitation-Based Simulation Activity to Create an Interprofessional Education Activity for Medical, Nursing, and Pharmacy Students
Source: MedEdPORTAL. 2020 Dec 11;16:11054. doi: 10.15766/mep_2374-8265.11054 (PMC7732132; doi:10.15766/mep_2374-8265.11054)
Supplement: Supplementary file 1 — Simulation Case Template.docxAgenda.docDebriefing Guide.docFaculty Training PowerPoint.pptxHospital Tech.docxMedication List.docxPrebrief Information.docxMedication Administration Record.docxFaculty Assessment Tool.xlsxStudent Questionnaire.docx [file mep_2374-8265.11054-s001.zip › H. Medication Administration Record.docx]

|  | | **Medication Administration Record (MAR)**  Name:___Patient from Team E_______________ Month:________ Year:  Allergies: _______MAGNESIUM SULFATE, IODINE_________________________ | | | |  |  |
| --- | --- | --- | --- | --- | --- | --- | --- |
| **Medication** | Time | | | | | | |
| Drug Name, Dosage, Route | **2pm 6pm** | | | | | | |
| Ipratropium 0.5mg/albuterol 2.5mg (DuoNeb®) nebulizations |  |  |  |  |  |  |  |
|  |  |  |  |  |  |  |  |
| Prescribed By: ED Doctor  Start: 1/30/18 End: 1/30/18 |  |  |  |  |  |  |  |
|  |  |  |  |  |  |  |  |
| Drug Name, Dosage, Route | **2pm** | | | | | | |
| Oxygen @ 2 LPM NC  Start: 1/30/18 End: _____ |  |  |  |  |  |  |  |
|  |  |  |  |  |  |  |  |
| Prescribed By: ED Doctor |  |  |  |  |  |  |  |
|  |  |  |  |  |  |  |  |
| Drug Name, Dosage, Route |  | | | | | | |
| Start: End: _____ |  |  |  |  |  |  |  |
|  |  |  |  |  |  |  |  |
| Prescribed By: |  |  |  |  |  |  |  |
|  |  |  |  |  |  |  |  |
| Drug Name, Dosage, Route |  | | | | | | |
| Start: End: _____ |  |  |  |  |  |  |  |
|  |  |  |  |  |  |  |  |
| Prescribed By: |  |  |  |  |  |  |  |
|  |  |  |  |  |  |  |  |
|  | | |  |  |  | |  |
| **NOTES:** | | | **Signature** | **Initial** | Signature | | Initial |
| **Meds Prior to Admission:** HCTZ 50mg QD,  Albuterol MDI 2 inh Q4-6hr prn,  Advair Diskus 250/50 BID | | | ED Pharmacist |  |  | |  |
|  | | |  |  |  | |  |
|  | | |  |  |  | |  |

| SIGNATURE | INITIALS | SIGNATURE | INITIALS | SIGNATURE | INITIALS |
| --- | --- | --- | --- | --- | --- |
|  |  |  |  |  |  |
|  |  |  |  |  |  |
|  |  |  |  |  |  |
|  |  |  |  |  |  |
