# Supplementary material for: Using a Resuscitation-Based Simulation Activity to Create an Interprofessional Education Activity for Medical, Nursing, and Pharmacy Students
Source: MedEdPORTAL. 2020 Dec 11;16:11054. doi: 10.15766/mep_2374-8265.11054 (PMC7732132; doi:10.15766/mep_2374-8265.11054)
Supplement: Supplementary file 1 — Simulation Case Template.docxAgenda.docDebriefing Guide.docFaculty Training PowerPoint.pptxHospital Tech.docxMedication List.docxPrebrief Information.docxMedication Administration Record.docxFaculty Assessment Tool.xlsxStudent Questionnaire.docx [file mep_2374-8265.11054-s001.zip › J. Student Questionnaire.docx]

***Please choose your discipline:* Nursing Pharmacy Physician**

***TEAM Performance***

Following your participation in the Interprofessional activity, please rate your team’s performance on the following items.

|  | | Strongly Agree  (SA) | Agree  (A) | Disagree  (D) | Strongly Disagree  (SD) | Not Applicable  (NA) |
| --- | --- | --- | --- | --- | --- | --- |
| *Values/Ethics Components* | | | | | | |
| 1. | **Respected the expertise of other health professionals** | SA | A | D | SD | NA |
| 2. | **Worked well with members of the healthcare team** | SA | A | D | SD | NA |
| 3. | **Established trust and rapport with patients, families and team member** | SA | A | D | SD | NA |
| *Roles and Responsibilities Components* | | | | | | |
| 4. | **Engaged other healthcare professionals in the evaluation and treatment process** | SA | A | D | SD | NA |
| 5. | **Utilized abilities of team members to optimize patient care** | SA | A | D | SD | NA |
| *Communication Components* | | | | | | |
| 6. | **Used effective communication tools and techniques** | SA | A | D | SD | NA |
| 7. | **Provided information in a form that is understandable to non-healthcare professionals** | SA | A | D | SD | NA |
| 8. | **Interacted in a respectful manner when dealing with conflict** | SA | A | D | SD | NA |
| *Teamwork Components* | | | | | | |
| 9. | **Engaged the patient in discussions/decisions about his/her care** | SA | A | D | SD | NA |
| 10. | **Demonstrated leadership practices which facilitated effective teamwork** | SA | A | D | SD | NA |
| 11. | **Performed effectively as a team by participating in a variety of roles** | SA | A | D | SD | NA |
| *Self-Evaluation Components*  Rate your performance on the following items. | | | | | | |
| 12. | **Recognized how individual knowledge, skills, and experience and the hierarchy of the team contributed to effective collaborative care** | SA | A | D | SD | NA |
| 13. | **Performed discipline specific skills and tasks well** | SA | A | D | SD | NA |
| 14. | **Used strategies to increase effectiveness of team-based care** | SA | A | D | SD | NA |
| 15. | **Do you feel like this simulation has helped prepare you for real situations you may face in the future?** | SA | A | D | SD | NA |

*Activity Feedback*

| 16. | Overall, this was a well-designed, realistic simulation activity | SA | A | D | SD | NA |
| --- | --- | --- | --- | --- | --- | --- |

**Do you have any suggestions, pros and/or cons, that could add to the improvement of this simulation in years to come? What could be done/not done to improve this activity?**

____________________________________________________________________________________________________________________________________________________________________________________________________________________________________________________________________________________________________________________________________________________________________________________________________________________________________________________________________________________________________________________________________________________________________________________________________________

**Additional comments:**

____________________________________________________________________________________________________________________________________________________________________________________________________________________________________________________________________________________________________________________________________________________________________________________________________________________________________________________________________________________________________________________________________________________________________________________________________________
