# Supplementary material for: Using a Resuscitation-Based Simulation Activity to Create an Interprofessional Education Activity for Medical, Nursing, and Pharmacy Students
Source: MedEdPORTAL. 2020 Dec 11;16:11054. doi: 10.15766/mep_2374-8265.11054 (PMC7732132; doi:10.15766/mep_2374-8265.11054)
Supplement: Supplementary file 1 — Simulation Case Template.docxAgenda.docDebriefing Guide.docFaculty Training PowerPoint.pptxHospital Tech.docxMedication List.docxPrebrief Information.docxMedication Administration Record.docxFaculty Assessment Tool.xlsxStudent Questionnaire.docx [file mep_2374-8265.11054-s001.zip › B. Agenda.doc]

**Interprofessional Crisis Management**

**Suggested Timeline or Agenda**

| Time | Duration | Agenda item | Notes |
| --- | --- | --- | --- |
| 9:00 | 15 min | Sign-in/arrival (classroom) |  |
| 9:15 | 10 min | Introduction and tour of sim room |  |
| 9:25 | 10 min | Pre-brief begins |  |
| 9:35 | 20 min | Activity begins |  |
| 9:55 | 25 min | Activity ends; debrief begins |  |
| 10:20 | 10 min | Debrief ends; students complete evaluations and are then dismissed |  |
| 10:30 | 15 min | Sign-in/arrival (classroom) |  |
| 10:45 | 10 min | Introduction and tour of sim room |  |
| 10:50 | 10 min | Pre-brief begins |  |
| 11:00 | 20 min | Activity begins |  |
| 11:20 | 25 min | Activity ends; debrief begins |  |
| 11:45 | 10 min | Debrief ends; students complete evaluations and are then dismissed |  |
| 12:00 | 60 minutes | Lunch Break, Restock as needed, Team Transfers, Etc. |  |
| 1:00 | 15 min | Sign-in/arrival (classroom) |  |
| 1:15 | 10 min | Introduction and tour of sim room |  |
| 1:25 | 10 min | Pre-brief begins |  |
| 1:35 | 20 min | Activity begins |  |
| 1:55 | 25 min | Activity ends; debrief begins |  |
| 2:20 | 10 min | Debrief ends; students complete evaluations and are then dismissed |  |
| 2:30 | 15 min | Sign-in/arrival (classroom) |  |
| 2:45 | 10 min | Introduction and tour of sim room |  |
| 2:50 | 10 min | Pre-brief begins |  |
| 3:00 | 20 min | Activity begins |  |
| 3:20 | 25 min | Activity ends; debrief begins |  |
| 3:45 | 10 min | Debrief ends; students complete evaluations and are then dismissed |  |
| 4:00 | 30 min | Faculty Debrief |  |
| 4:30 | 30 min | Break down, Clean up, Dismiss |  |
