# Supplementary material for: Using a Resuscitation-Based Simulation Activity to Create an Interprofessional Education Activity for Medical, Nursing, and Pharmacy Students
Source: MedEdPORTAL. 2020 Dec 11;16:11054. doi: 10.15766/mep_2374-8265.11054 (PMC7732132; doi:10.15766/mep_2374-8265.11054)
Supplement: Supplementary file 1 — Simulation Case Template.docxAgenda.docDebriefing Guide.docFaculty Training PowerPoint.pptxHospital Tech.docxMedication List.docxPrebrief Information.docxMedication Administration Record.docxFaculty Assessment Tool.xlsxStudent Questionnaire.docx [file mep_2374-8265.11054-s001.zip › C. Debriefing Guide.doc]

**Interprofessional Crisis Management**

**Faculty Debriefing Guide**

**IPE Objectives Specific to this Activity:**

- VE4 Respect the unique cultures, values, roles/responsibilities, and expertise of other health professions
- RR6 Communicate with team members to clarify each member’s responsibility in executing components of a treatment plan or public health intervention. (Closed loop communication and assigned tasks).
- CC6 Use respectful language appropriate for a given difficult situation, crucial conversation, or interprofessional conflict (communicate in a high stress situation, communication to family and each other as the case escalated).
- TT8 Reflect on individual and team performance for individual, as well as team, performance improvement.

**Debrief Overview**

I. Open the debriefing discussion with a brief recap of the scenario. (1-2 min)
II. Discuss the experience of the clinical event. (5 minutes)
III. Discuss experiences with the planning portion of the encounter (1-3 min)
IV. Discuss the experience of the encounter, including roles and interactions with different disciplines in the encounter (address stereotypes or controversial comments that may have come up during the session) (10-12 minutes)
V. Future Strategies (If you need to flex, take time from this section-otherwise 3 min)

**Suggestions:**

*If students provide an answer to a question while answering another question, you do not need to repeat the question. However, please note that the items in red are specific to the assessment and must be asked.*

Before beginning the debriefing, any yellow cards should be given to the clinician on the team to discuss with the individual student or team as needed.

I. Open the debriefing discussion (1-2 minutes)

Brief introductions and ask the team for their impression of the activity.

*“Okay everyone. That was an interesting session. Please tell me, in your opinion, how did it go?”*

The students are given a chance to express their immediate thoughts and feelings. Usually, they will focus on their individual performance. Let them vent briefly. This is the where students may say things like: “*I can’t believe I did….”* Or, “*I think that went pretty well.”*

II. Discuss the experience of the clinical event (5 minutes)

Students find it hard to move forward until they have closure on their performance and any clinical questions about the case. Therefore, a clinical faculty (either MD or APN) will debrief immediate concerns. Try to limit this to 5 minutes or less as it can quickly consume all of the time reserved for the IPE debrief.

III. Discuss the experience of the planning session (1-3 minutes)

*Say: “At the beginning of the activity, you were given the opportunity to meet each other, review the scenario, walk through of the resuscitation bay and familiarize yourself with the equipment. In addition, you were had a chance to discuss your role and plan how you would prepare for the encounter. As you think about the planning…:”*

1. ***What do you think were the goals and objectives of this simulation? (Or, “Putting it in your own words, what was the purpose of this simulation?)***
2. ***Did you coordinate any of the tasks or procedures? (Or, “If you were a fly on the wall, could you identify who would do what jobs?”)***
3. ***Did you have a good understanding of the roles and responsibilities for each member? (Or, “Did you have to ask questions about what your teammates could and could not do clinically?”)***
4. ***What challenges or potential problems did you identify? (Or, “What were your fears and concerns?”)***

*(Optional question if time allows) After having experienced this scenario what changes would you make to your* ***planning*** *session?)*

IV. Discuss the experience of the encounter (10-12 minutes) (**Note questions 2, 3 and 4 MUST be asked)*

*Say: “Moving on to the actual encounter, we would like you to share your overall experiences.”*

1. ***Were your interactions with one another different from what you expected going in? If so how?***
2. ***Can you share examples of the things your team did effectively (or what were the successes)?* MUST ASK THIS QUESTION***
3. ***Were there any difference(s) in perceptions of the situation(s) or event(s)? Or, “Did your team have a shared mental model about procedures, roles and responsibilities and the connections between them?* If not, why not? MUST ASK THIS QUESTION***
4. ***Was the communication effective? For example, was there situational awareness and was it communicated? Did members use closed loop communication tools such as call out and check back? Was there psychological safety and/or a willingness to admit mistakes?* MUST ASK THIS QUESTION***
5. ***What challenges, barriers or problems were experienced by either the team or individual members? (Individual challenges should focus on the problem, NOT the person.)***
6. ***Were your solutions effective? (Or did the team develop effective AND innovative solutions?)***

V. Future Strategies (1-3 minutes)

*Think about your take home message from today and other kinds of inter-professional work.”*

1. ***What did you find that you have in common that you did not anticipate?***
2. ***What will you take with you to improve team performance in the future?***
3. ***Any additional thoughts or comments?***
VI. Yellow cards and wrap up (as needed).

*Significant clinical concerns should be addressed by the clinician at this time. If the issue is related to the team, discuss with all students present. If it is related to a specific student, discreetly remove the student and remediate. .*

*If no yellow cards say: “Thank you for your active participation and feedback today! Please complete the questionnaire before leaving!”*

**In addition to the IPE Goals and Objectives listed at the beginning of the guide, this activity may also be considered in partial fulfillment of the following discipline specific goals and/or objectives:**

**Nursing Specific Goals or Objectives:**

1. Deliver nursing care focused on caring and competency in professional knowledge, skills and values for culturally diverse populations across the life span and health continuum.

2. Exhibit professional and personal responsibility and accountability in provision of nursing care

3. Demonstrate critical thinking and clinical decision making in the delivery, coordination and collaboration of nursing care.

4. Utilize effective systematic inquiry based on research and best evidence to provide safe patient centered care and continuous quality improvement

5. Incorporate effective communication, collaboration and advocacy in working with patients, families, groups, communities and the health care team for improving health care outcomes.

6. Demonstrate beginning leadership and patient-centered care in a health care system that is influenced by health policy, finance and regulatory environment.

**Pharmacy Specific Goals or Objectives:**

1. Provide appropriate patient-centered care (Patient-Centered Care)

- Interpret patient information, physical assessment, and laboratory values to develop a prioritized therapeutic plan of care
- Design and implement a therapeutic plan of care based on patient information, health care setting, evidence-based guidelines and current health information literature
- Monitor and adjust a therapeutic plan of care to achieve optimal health outcomes
- Evaluate, alleviate, and prevent medication-related problems

2. Exhibit professionalism and work as an effective team member (Professionalism

and Teamwork)

- Collaborate with other professionals to achieve a shared goal, optimize care, and/or advance learning
- Demonstrate accountability and professional behavior
- Recognize individual roles, limitations and expertise to form a highly functioning team
- Employ ethical and legal principles

**Medicine Specific EPA Goals/Objectives:**

**EPA 9** – Collaborate as a member of an interprofessional team. Their functions included:

- Identify team roles and responsibilities
- Establish/maintain climate of respect and trust
- Communicate with and include team members
- Use attentive listening skills
- Understand one’s own roles and limits and seek help
- Help team members in need

**EPA 10** – recognizing patient requiring urgent or emergent care and initiate evaluation and management. Their functions included:

- Recognize abnormal VS
- Recognize severity of illness
- Start initial care plan for decompensating patient
- Engage team members for required immediate response
- Understand initiating code response and participating as a team member
